# Microscopy examination of red blood and yeast cell agglutination induced by bacterial lectins

**DOI:** 10.1371/journal.pone.0220318

**Published:** 2019-07-25

**Authors:** Jana Mrázková, Lenka Malinovská, Michaela Wimmerová

**Affiliations:** 1 Department of Biochemistry, Faculty of Science, Masaryk University, Brno, Czech Republic; 2 National Centre for Biomolecular Research, Faculty of Science, Masaryk University, Brno, Czech Republic; 3 Central European Institute of Technology, Masaryk University, Brno, Czech Republic; University of Liverpool, UNITED KINGDOM

## Abstract

Lectins are a group of ubiquitous proteins which specifically recognize and reversibly bind sugar moieties of glycoprotein and glycolipid constituents on cell surfaces. The mutagenesis approach is often employed to characterize lectin binding properties. As lectins are not enzymes, it is not easy to perform a rapid specificity screening of mutants using chromogenic substrates. It is necessary to use different binding assays such as isothermal titration calorimetry (ITC), surface plasmon resonance (SPR), microscale thermophoresis (MST), enzyme-linked lectin assays (ELLA), or glycan arrays for their characterization. These methods often require fluorescently labeled proteins (MST), highly purified proteins (SPR) or high protein concentrations (ITC). Mutant proteins may often exhibit problematic behaviour, such as poor solubility or low stability. Lectin-based cell agglutination is a simple and low-cost technique which can overcome most of these problems. In this work, a modified method of the agglutination of human erythrocytes and yeast cells with microscopy detection was successfully used for a specificity study of the newly prepared mutant lectin RS-IIL_A22S, which experimentally completed studies on sugar preferences of lectins in the PA-IIL family. Results showed that the sensitivity of this method is comparable with ITC, is able to determine subtle differences in lectin specificity, and works directly in cell lysates. The agglutination method with microscopy detection was validated by comparison of the results with results obtained by agglutination assay in standard 96-well microtiter plate format. In contrast to this assay, the microscopic method can clearly distinguish between hemagglutination and hemolysis. Therefore, this method is suitable for examination of lectins with known hemolytic activity as well as mutant or uncharacterized lectins, which could damage red blood cells. This is due to the experimental arrangement, which includes very short sample incubation time in combination with microscopic detection of agglutinates, that are easily observed by a small portable microscope.

## Introduction

Sugars in various forms occur in all organisms, where they have a wide range of functions. They play an irreplaceable role as sources of energy as well as constitutive components of cell walls. Saccharides often form inseparable parts of various proteins and lipids. The glycoproteins and glycolipids present on cell surfaces can bear terminal oligosaccharides which are readily accessible. These sugar moieties are recognized and bound by a special class of proteins called lectins [1].

Lectins are neither antibodies nor enzymes, and they also differ from proteins that are specialized for the transport of free saccharides. They bind a wide range of carbohydrates reversibly and with a very high specificity, therefore they participate in recognition and interactions at the cell level. As they are ubiquitous, their precise function also depends on the organism in which they occur or their localization in a particular tissue. Lectins are involved in many biological processes, for example cell development and migration, immune defence, plant-microbial symbiosis, endocytosis, phagocytosis or fertilization [2]. Lectins produced by pathogens are involved in the recognition of host cells and subsequent adhesion to the cell surface during the initial step of infection, and therefore lectins are often counted as virulence factors [3].

Due to their ability to bind a wide range of saccharides with high selectivity, lectins have great potential for use in biotechnologies. They are utilized in biosensors for detecting specific markers related to a particular disease [4]. Labeled lectins can serve for histochemical analyses to investigate changes in sugar patterns exhibited on the surfaces of cells and tissues that are often associated with cancer development [5]. Lectins have a potential in treatment of immune response-related diseases and in healing of wounds or in anticancer therapy. Many lectins also exhibit antimicrobial or antiparasitic activities, and some of them also have an insecticidal effect. Currently, lectins are being tested as parts of carbohydrate-based drug-delivery systems [6] and they are used for the development of novel glycomimetic inhibitors, which can be used for the treatment of diseases [7]. All these facts show why it is so important to study lectin-sugar interactions.

The mutagenesis approach is used for the elucidation of sugar binding mechanisms of lectins [8] as well as for the modification of lectin properties [9, 10]. The functional characterization of lectins and their mutant variants is performed by methods such as isothermal titration calorimetry, surface plasmon resonance, enzyme-linked lectin assay, microscale thermophoresis, or glycan arrays [11–13]. These methods may require samples of high purity, high concentration, immobilized ligands or labeled proteins. Therefore, it is difficult to routinely utilize these methods in many cases, especially when proteins exhibit problematic behaviour such as poor solubility or low stability with a tendency to aggregation. In addition, protein production and purification are not often straightforward, and may result in low protein yields or proteins with decreased or no activity. All these problems can even be made worse when preparing mutant variants of a protein.

Agglutination assay is a method based on cell agglutination caused by lectins. Multivalent lectins specifically recognize and bind sugar moieties present on the surface of particular types of cells (e.g. erythrocytes) which leads to cross-linking of the cells and a formation of cell clumps called agglutinates [2]. This easy and inexpensive method is often utilized for various purposes. The ability of lectins to selectively agglutinate red blood cells is utilized for human and animal blood group typing and subtyping [14–16]. Hemagglutination (HA) or yeast agglutination assay (YA) is used to search for new lectins of various organisms [17, 18] as well as for the rapid testing of lectin activity [19]. The agglutination of microbial cells often serves for studying the interactions between lectins and pathogens [20]. Hence, it helps to reveal the role of lectins in innate immunity [21] and in self-defence against bacteria and fungi [22, 23]. In addition, lectin-mediated cell agglutination can be employed for the identification of different microbial species. Using a panel of diverse lectins, which selectively recognize the glycans present on the cell surfaces, enables the differentiation of organisms which are taxonomically very close [24, 25].

Agglutination inhibition (AI) assay is routinely utilized for investigating lectin sugar-binding specificity. In this method, various sugars are used to find the saccharides that are able to inhibit the process of cell agglutination, which means that they are specifically bound by a particular lectin. All saccharides are serially diluted to determine the lowest concentration of each sugar that is still able to inhibit the agglutination. Therefore, this assay can also serve for a semi-quantitative determination of the lectin’s affinity [26].

Agglutination (inhibition) assays were effectively utilized for testing agglutination activity and for specificity studies of non-purified lectins in homogenized mycelium [17], fish serum [21], shrimp plasma [27] or fish skin mucus [28]. Also, it was confirmed that this method can be used for testing recombinant lectins, which were produced by *E*. *coli*, directly in the crude bacterial cytoplasmic extracts without any preceding purification [26]. Hence, this assay could be employed to study and determine the sugar-binding properties of mutant lectins with problematic behaviour.

Standard protocol involves performing the HA or hemagglutination inhibition assay (HI) in a 96-well microtiter plate where a lectin or a sugar is serially diluted and then mixed with constant concentration of red blood cell (RBC) suspension (HA) or constant concentration of a lectin sample and RBC suspension (HI). The plate is incubated at room temperature for 1 to 2 hours and the assay is visually inspected. The difference between agglutination (diffuse reddish mat) and sedimentation of non-agglutinated erythrocytes (red dot) is manually interpreted [29]. Considerable volume of lectin sample can be consumed because up to 50 μl of the sample is pipetted into each well when HI is performed. In this work, the method of cell agglutination with microscopic detection was successfully optimized and utilized for determining the specificity change of the *Ralstonia solanacearum* mutant lectin RS-IIL_A22S. Wild-type RS-IIL [30] belongs to the PA-IIL lectin superfamily as well as lectins PA-IIL from *Pseudomonas aeruginosa* [31], CV-IIL from *Chromobacterium violaceum* [32], BC2L-A [33] and the C-terminal domain of lectin BC2L-C [34] from *Burkholderia cenocepacia*. All these lectins are sequential and structural homologues. Although they bind saccharides with similar arrangement, they differ in their preferences towards these saccharides. The sugar preference of each lectin is determined by three consecutive amino acids which form the so-called “specificity binding loop”. The first amino acid of this loop is crucial for lectin specificity. It was demonstrated that the replacement of the serine in the fucose-preferring lectin PA-IIL with alanine, which is in the same position in the mannose-preferring lectin RS-IIL, led to a change in the PA-IIL sugar preference from fucose to mannose [35]. To determine the sensitivity of AI to small differences in lectin specificities, and to optimise the procedure, the CV-IIL lectin was used. It is a tetramer of molecular weight 47.3 kDa bearing one binding site per monomer. Previous results of ELLA experiments showed that the CV-IIL preference for d-mannose is only 6-fold weaker than that for l-fucose, and therefore it can be described as a fucose/mannose-binding lectin [36]. Therefore, it was selected as a good candidate for probing the ability of this method to determine subtle differences in lectin specificity. Human red blood cells and yeast cells were used in agglutination assays, because their cell surface glycans contain terminal l-fucose and d-mannose, respectively. Both types of cells are commonly utilized for the determination of lectin specificity [19, 27] and are readily available. In addition, a simple portable bright-field microscope can be used for the observation of agglutinates.

Furthermore, the one-point mutant RS-IIL_A22S was prepared. This mutation was expected to cause a specificity change of the lectin from mannose to fucose. However, this reverse substitution replacing Ala with Ser has never been performed experimentally.

SPR and ITC using purified proteins gave similar results comparable to those obtained from simple cell agglutination assays using RBC and yeast cells. Also, the results of the agglutination method with microscopic detection were concordant with results obtained by agglutination (inhibition) assays performed in a 96-well microtiter plate according to the standard protocol, which means that the method was validated. Moreover, the same data were even obtained using crude bacterial cytoplasmic extracts without the need to further purify the proteins.

## Materials and methods

### Production and purification of lectins CV-IIL and RS-IIL

Lectins CV-IIL and RS-IIL were produced as previously described [30, 36]. *Escherichia coli* Tuner (DE3) cells harbouring plasmid with the gene for CV-IIL or *Escherichia coli* BL21 (DE3) cells harbouring plasmid with the gene for RS-IIL were cultured in LB broth medium containing ampicillin (100 μg/ml) at 37°C until the OD_600 nm_ of the cell culture reached 0.6. Lectin production was induced by adding isopropyl β-d-1-thiogalactopyranoside (IPTG) to a final concentration of 0.5 mM. Cell culture was incubated at 30°C for 3 hours and then harvested and resuspended in a loading buffer (20 mM Tris/HCl, 150 mM NaCl, 100 μM CaCl_2_, pH 7.5). Cells were disintegrated by sonication (Vibra-Cell, Sonics) and the cytosolic fraction containing soluble proteins was separated by centrifugation at 21,000 g and 4°C for 1 hour. The lectins were purified by affinity chromatography (ÄKTAFPLC, GE Healthcare) on a d-mannose-agarose column and eluted with an elution buffer containing d-mannose (20 mM Tris/HCl, 150 mM NaCl, 100 μM CaCl_2_, 100 mM d-mannose, pH 7.5). The purified lectins were dialyzed against distilled water for one week, immediately freeze-dried and stored at -20°C.

### Mutagenesis, production and purification of RS-IIL_A22S

Previously prepared plasmid pET-25(b+)_*rs2l* containing the gene for RS-IIL served as a template for the site-directed mutagenesis via amplification of the whole plasmid using a pair of complementary primers carrying the desired base pair substitutions. The reaction mixture (50 μl in total) consisted of 5 μl 10× *PfuUltra* HF reaction buffer (Agilent Technologies), 1 μl dNTP mix (10 mM each nucleotide, Sigma), 1 μl of each primer (concentration of each primer: 100 μM; primer *rs2L-A22S* 5'-CTT CGG TGT GAC GGC ATT TGC AAA TAG CGC GAA CAC CCA GA-3', primer *rs2L-A22S-anti* 5'-TCT GGG TGT TCG CGC TAT TTG CAA ATG CCG TCA CAC CGA AG-3', 41-mers), 1 μl of the plasmid pET-25(b+)_*rs2l* (100 ng/μl) and 1 μl *PfuUltra* HF DNA polymerase (2.5 U/μl; Agilent Technologies). The reaction conditions were: 1) 95°C 2 min, 2) 18 cycles: 95°C 50 s, 65°C 50 s, 72°C 12 min, 3) 72°C 10 min. Subsequently 1 μl of *Dpn* I restrictase (20,000 U/ml, New England BioLabs) was added to the reaction mixture and the mixture was incubated at 37°C for 3 hours. *E*. *coli* Tuner (DE3) cells were transformed by the digested reaction mixture and the introduced mutation was confirmed by DNA sequencing. Mutant RS-IIL_A22S was produced and purified as described above for CV-IIL and RS-IIL. The purified protein was intensively dialyzed against 200 mM ammonium hydrogen carbonate for one week, freeze-dried and stored at -20°C.

### Surface plasmon resonance (SPR) measurement

SPR measurements were performed on BiaCore 3000 (GE Healthcare) at 25°C using immobilized monosaccharides α-d-galactopyranoside, α-d-mannopyranoside and α-l-fucopyranoside. HEPES buffer (10 mM HEPES, 150 mM NaCl, 0.005% TWEEN 20, 2 mM CaCl_2,_ pH 7.4) was used as a running buffer. Surface of a sensor chip CM4 (GE Healthcare) was coated with streptavidin using a standard amine coupling method. Briefly, the carboxymethylated dextran matrix was activated with an N-hydroxysuccinimide/N-(3-dimethylaminopropyl)-N'-ethylcarbodiimide hydrochloride solution (NHS/EDC, GE Healthcare) and then streptavidin in 10 mM sodium acetate (pH 5.0) in a concentration of 100 μg/ml was injected onto the chip. Unreacted groups were blocked with 1 M ethanolamine/HCl (pH 8.5). The volume of used solution was 50 μl for each channel and a flow rate was 5 μl/min. Monosaccharides bound to the biotinylated polyacrylamide (biotin-PAA-monosaccharide, Lectinity, Russia) were used for the immobilization. Each biotin-PAA-monosaccharide was diluted in the running buffer to a concentration of 200 μg/ml. 50 μl of this solution was injected onto one particular channel at a flow rate of 5 μl/min.

Lectin CV-IIL or wild-type RS-IIL or mutant RS-IIL_A22S was dissolved in the working buffer (10 mM HEPES, 150 mM NaCl, 0.005% TWEEN 20, 2 mM CaCl_2_, pH 7.4) and incubated overnight at room temperature. Next day, the sample was centrifuged for 2 minutes at 16,000 g and the supernatant was subsequently filtered using 0.2 μm centrifugal filter (VWR International). The concentration of the lectins was determined spectrophotometrically using NanoDrop ND-1000 Spectrophotometer (Thermo Scientific). Each lectin was diluted in the working buffer to a concentration of 100 μg/ml. 20 μl of each protein sample was injected onto the CM4 chip with immobilized carbohydrates at a flow rate of 5 μl/min. The sensor chip was washed twice with 100 mM EDTA after each protein measurement. The channel with immobilized α-d-galactopyranoside was used as a blank.

### Isothermal titration calorimetry (ITC) measurement

Lectin CV-IIL or mutant RS-IIL_A22S was dissolved in a buffer (100 mM Tris/HCl, 0.5 mM CaCl_2_, pH 7.5) and incubated overnight at room temperature. Next day, the sample was centrifuged, filtered and the concentration was determined. All experiments were performed using the iTC_200_ microcalorimeter (Malvern Instruments) at 25°C. 20 aliquots of 2 μl of l-fucose or d-mannose dissolved in the same buffer were automatically added at 4 minute interval to the protein solution present in the calorimeter cell. The CV-IIL concentration in the cell was 0.181 mM when titrated by l-fucose (the concentration in the syringe was 2.013 mM) and 0.152 mM when titrated by d-mannose (the concentration in the syringe was 2.32 mM). The RS-IIL_A22S concentration was 0.065 mM and 0.165 mM, respectively, when titrated by l-fucose (0.7 mM) and d-mannose (3.48 mM), respectively. The concentrations were calculated with MW of CV-IIL monomer (11.84 kDa) and RS-IIL_A22S monomer (11.62 kDa). Integrated heat effects were analyzed using Microcal Origin 7 software (Malvern Instruments).

### Preparation of RBC and yeast cell suspensions

Anonymized human blood (2 ml, blood group 0^+^) treated with natrium citrate was obtained from the Transfusion and Tissue Department of The University Hospital in Brno. The blood was centrifuged at 2000 g for 3.5 minutes, supernatant was discarded, and the cell pellet was washed three times with PBS buffer (137 mM NaCl, 2.7 mM KCl, 8 mM Na_2_HPO_4_, 1.5 mM KH_2_PO_4_, pH 7.4). RBC were treated with 0.1% papain for 1 hour at 37°C and again washed three times with PBS buffer. RBC pellet was diluted to 50% (v/v) with PBS buffer, stabilized with 0.01% natrium azide and stored at 4°C. The life span of RBC was up to three weeks. 0.5 g of fresh baker’s yeast (Uniferm, Germany) was resuspended in 0.5 ml of the working buffer (10 mM HEPES, 150 mM NaCl, 0.005% (v/v) TWEEN 20, 2 mM CaCl_2_, pH 7.4) and stored at 4°C. The yeast suspension was freshly prepared before each agglutination assay. The same batch of each cell suspension was used for the agglutination assay and subsequent agglutination inhibition assays.

### Optimization of cell concentration, incubation time and sample volume on the glass slide used in the agglutination assay

RBC and yeast cell suspensions were diluted to three different concentrations (5%, 10% and 20%) with working buffer. Lectin CV-IIL was dissolved in working buffer (10 mM HEPES, 150 mM NaCl, 0.005% (v/v) TWEEN 20, 2 mM CaCl_2_, pH 7.4) and incubated overnight at room temperature. Next day, the sample was centrifuged, filtered and the concentration was determined. Whole lectin tetramer was considered as one function unit therefore all concentrations were calculated with MW of tetramer (47.31 kDa). The lectin in a concentration of 0.1 mM was serially diluted with working buffer. 20 μl of the cell suspension with desired concentration was mixed in a 1.5 ml microcentrifuge tube with 20 μl of lectin sample thoroughly and reproducibly. The mixture was incubated for 5 minutes (RBC and yeast cell agglutination) or 10 minutes (yeast cell agglutination only) at room temperature and mixed again. Samples of the mixture in three volumes 20 μl, 10 μl and 5 μl for each concentration were applied to microscope glass slides and carefully covered by microscope cover glass. Agglutination was observed using a small portable bright-field microscope Levenhuk D2L NG (Levenhuk). Set-up with objective magnification 4×, lower illumination and fully open diaphragm was used. Images were captured using a microscope digital camera DEM135 (Levenhuk) with the software ToupView for Windows (Levenhuk). The total magnification including the objective (4×) and the camera (32×) contributions was 128×. Whole sample on the glass slide was inspected and representative images were taken. This procedure was performed with all lectin samples from serial dilution. 20 μl of the working buffer instead of the lectin sample was used as a negative control.

### Determination of lectin concentration appropriate for agglutination inhibition assays

Different batches of the CV-IIL lectin or the RS-IIL_A22S mutant were dissolved in the working buffer and incubated overnight at room temperature. Next day, the sample was centrifuged, filtered and the concentration was determined. All concentrations were calculated with MW of tetramers. Lectin CV-IIL or mutant RS-IIL_A22S (MW of tetramer is 46.42 kDa) in a concentration of 0.1 mM was serially diluted with working buffer. 10 μl of 5% RBC or yeast cell suspension was mixed in a 1.5 ml microcentrifuge tube with 10 μl of lectin sample. The mixture was incubated for 5 minutes in the case of hemagglutination and 10 minutes in the case of yeast cell agglutination, respectively, at room temperature. The mixture was mixed again and 10 μl of the mixture was applied to the microscope glass slide and carefully covered by microscope cover glass. Agglutination was observed and images were captured as described above. This procedure was performed with all lectin samples from serial dilution. 10 μl of working buffer instead of lectin sample was used as a negative control.

### Agglutination inhibition assays of purified lectins

Monosaccharides l-fucose and d-mannose were dissolved in sterile water (to concentration of 150 mM) and serially diluted in the working buffer to concentrations ranged from 20 mM to 1.25 μM. CV-IIL or RS-IIL_A22S were diluted to determined working concentrations (calculated with MW of tetramers). Lectin sample was mixed in microcentrifuge tube with the monosaccharide sample in 5 μl: 5 μl ratio. Hence, the final concentrations of ligand were two times lower. The mixture was incubated for 1 minute at room temperature. 10 μl of 5% RBC or yeast cell suspension was added immediately to the mixture after the incubation. The solution was mixed and incubated for additional 5 minutes (RBC) or 10 minutes (yeast cells) at room temperature. The mixture was mixed again and the sample of 10 μl was applied to the microscope glass slide and covered by cover glass. Agglutination was observed and images were captured as described above. This procedure was performed with all monosaccharide samples from serial dilution. The positive (mixture without monosaccharide) and negative control (mixture without lectin) experiments were performed in the same way as described. The corresponding volume of the working buffer was used instead of the omitted components.

### Agglutination inhibition assays of crude bacterial cytoplasmic extracts

CV-IIL, RS-IIL_A22S and wtRS-IIL were produced in *Escherichia coli* Tuner (DE3) cells. The cells were cultivated in 10 ml of LB broth medium with ampicillin. Lectin production was induced by adding IPTG and cultivation continued at 30°C for 3 hours. Cells were harvested and washed twice with the working buffer. Pellets were stored at -20°C. As a negative control, expressing *Escherichia coli* Tuner (DE3) cells without any plasmid were cultivated and processed in the same way as cells containing plasmids with genes for lectins. Before agglutination assay, each pellet was thawed and dissolved in 300 μl of the working buffer. Cells were then disrupted by sonication (Vibra-Cell, Sonics) and cytoplasmic extract was separated by centrifugation at 21,000 g and 4°C for 1 hour. Cytoplasmic extract was filtered using 0.2 μm centrifugal filter (VWR International), serially diluted with the working buffer and kept on ice. Determination of appropriate dilution of cytoplasmic extracts for subsequent agglutination inhibition assay was performed. Purified CV-IIL in a concentration of 0.1 mM was used as a positive control and the working buffer was used as a negative control. Hemagglutination and yeast agglutination inhibition assays were done in the same way as described above. Cytoplasmic extracts in determined dilution were used instead of purified lectins.

### Hemagglutination inhibition assays on microtiter plate

The CV-IIL lectin, the RS-IIL_A22S mutant and bacterial cytoplasmic extracts were prepared as described above. Each well (except the first well in the row) in a microtiter plate (U-bottomed 96-well microtiter plate, Anicrin) was filled with 50 μl of the working buffer. 50 μl of CV-IIL (25 μM, calculated as tetramer) or RS-IIL_A22S (50 μM, calculated as tetramer) was pipetted into the first well and serially diluted except for the last wells (controls). 50 μl of the RBC suspension was added to each well and mixed. 1%, 2% and 4% RBC suspensions were tested for CV-IIL, and 2% RBC suspension was used for RS-IIL_A22S. The plate was incubated for 1 hour at room temperature and the highest dilution of the lectin solution showing complete agglutination (the titer) was visually determined. Bacterial cytoplasmic extracts were processed and evaluated in the same way using 2% RBC suspension.

Monosaccharides l-fucose and d-mannose were prepared as described above. CV-IIL was prepared in titers 1, 2, 4 and 8. RS-IIL_A22S and cytoplasmic extracts was prepared in the titer 2. 25 μl of the working buffer was pipetted into each well except the first. 25 μl of the sugar in the working buffer were pipetted into the first well and serially diluted. Carbohydrates were used in concentrations 40 mM for CV-IIL, 8 mM for RS-IIL_A22S and cytoplasmic extract containing this lectin. 25 μl of the lectin/cytoplasmic extract was added to each well and mixed. For each road, a positive control (the buffer instead of a monosaccharide) and a negative control (the buffer instead of a lectin/extract) were done. The plate was incubated for 30 minutes at room temperature, then 2% RBC was added and the plate was incubated for additional 2 hours. The minimal inhibitory concentrations of carbohydrates were visually determined for each lectin or extract and compared.

### Influence of lectins on RBC fitness

CV-IIL or RS-IIL_A22S in concentrations of 0.1 mM (calculated as tetramers) were serially diluted with the working buffer. 50 μl of 5% RBC suspension was added to 50 μl of each diluted sample and mixtures were incubated at room temperature for 1 hour. 10 μl of the last dilution, which showed complete agglutination observed by the naked eye was transferred onto the glass slide and covered by the cover glass. The cell health fitness was observed using the motorized inverted fluorescence microscope IX81 (Olympus) and images were captured using the DP72 microscope digital camera (Olympus). 10 μl of the working buffer instead of the lectin sample was used as a negative control.

## Results and discussion

### Mutagenesis, production and purification of RS-IIL_A22S

In addition to the wild-type lectins CV-IIL and RS-IIL, the new RS-IIL_A22S mutant was prepared by *in vitro* site-directed mutagenesis. Amplification of a whole plasmid using a pair of complementary primers carrying the desired mutation was employed for the mutagenesis, and the replacement of serine with alanine at position 22 was confirmed by DNA sequencing. A typical yield of the purified mutant was only around 2.5 mg per litre of cell culture, which is lower than a typical yield of wild-type RS-IIL (wtRS-IIL), which was around 10 mg. The expression level of both proteins is generally much lower than for other recombinant lectins from the PA-IIL family [33, 36]. wtRS-IIL was already found to be less stable than CV-IIL or PA-IIL, and its functional characterization was difficult [36]. RS-IIL_A22S exhibited even worse solubility and decreased stability leading to a limitation in terms of its further characterization.

### Characterisation of lectin binding properties using SPR and ITC experiments

**Fig 1:**
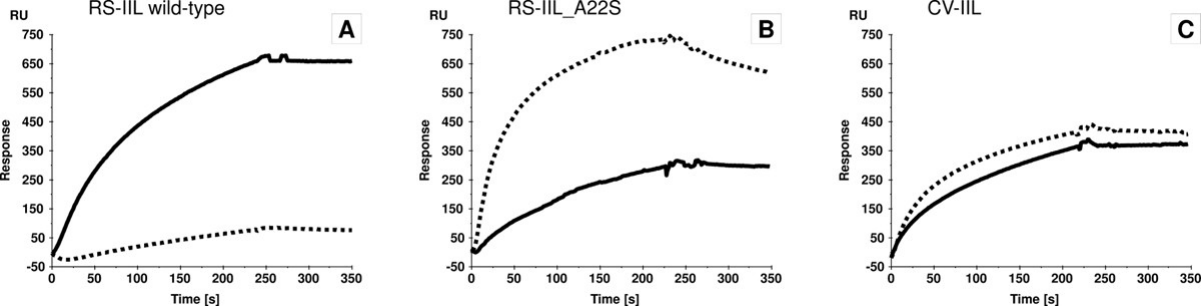
**Binding of wtRS-IIL (A), RS-IIL_A22S (B) and CV-IIL (C) on the sugar chip CM4 with immobilized sugars.** The response on the channel with immobilized α-d-mannopyranoside is shown as a solid-line curve, while the response on the channel with immobilized α-l-fucopyranoside is shown as a dotted-line curve. The differential curves were obtained after subtraction of the α-d-galactopyranoside channel.

In addition to the newly studied RS-IIL_A22S, CV-IIL was selected as an example of a lectin with almost the same specificity towards fucose and mannose. One of the methods for specificity determination is SPR. Using the same amount of different sugars immobilised on separate channels, the specificity of a lectin can be evaluated. SPR measurements of all three lectins were performed with immobilized α-d-mannopyranoside (PAA-Man) and α-l-fucopyranoside (PAA-Fuc), respectively, on the chip. The channel with α-d-galactopyranoside (PAA-Gal) served as a blank. While wtRS-IIL gave a much higher response on PAA-Man than on PAA-Fuc (Fig 1A), the responses of RS-IIL_A22S were the other way around (Fig 1B). SPR measurement of CV-IIL clearly showed that its specificities for PAA-Man and PAA-Fuc are very close (Fig 1C).

**Fig 2:**
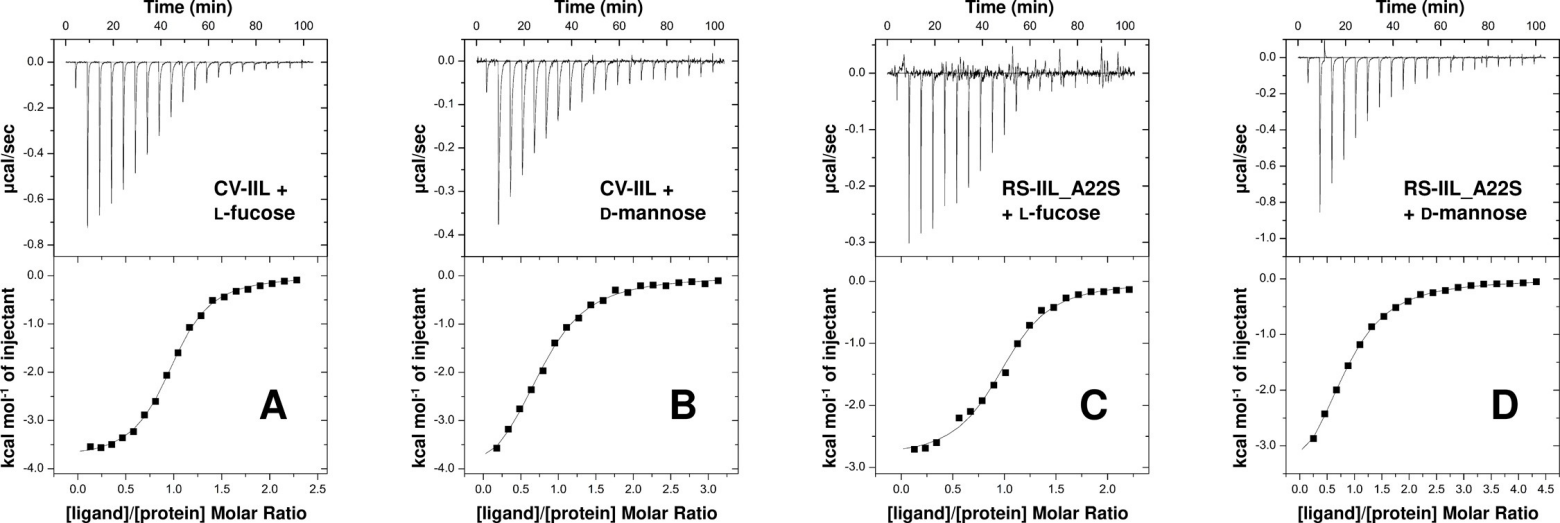
Isothermal titration calorimetry data. Titration of l-fucose (A) and d-mannose (B) to CV-IIL, respectively, and l-fucose (C) and d-mannose (D) to RS-IIL_A22S. Upper panels: Data obtained from 20 automatic injections (2 μl) of the sugar into the protein-containing cell. Lower panels: Plot of total heat released as a function of ligand/protein molar ratio for titrations shown in upper panels. The solid line represents the best fit for the obtained data.

Microcalorimetry experiments using ITC were further performed to quantitatively determine the equilibrium dissociation constants (K_D_) of the RS-IIL_A22S mutant and CV-IIL towards free monosaccharides, l-fucose (Fuc) and d-mannose (Man) (Fig 2). K_D_ of RS-IIL_A22S for Fuc and Man was 3.8 × 10^−6^ M and 46.5 × 10^−6^ M, respectively. The difference in affinities is over 12-fold. The micromolar affinity of the mutant to fucose showed that the point substitution led to a change in specificity but did not significantly affect its affinity [36]. The K_D_ of CV-IIL for Fuc and Man was 8.2 × 10^−6^ M and 25.6 × 10^−6^ M, respectively, exhibiting only a 3-fold higher affinity of this lectin towards fucose than mannose. This ratio corresponds to the ratio obtained by ELLA experiments, which were done for both these ligands previously [36].

The ITC and SPR results clearly confirmed the predicted switch in RS-IIL_A22S sugar preference from d-mannose to l-fucose compared to wtRS-IIL caused by a single point substitution of the first amino acid in the specificity binding loop, and emphasises the importance of this residue for lectins’ specificities in the PA-IIL family [30, 35, 37]. Unfortunately, this conclusion required a number of time-demanding steps, including purification and binding characterization of the produced proteins. Mutagenesis is frequently employed for detailed investigation of lectin properties and/or the preparation of new lectins with novel binding preferences. This is often done using directed-evolution methods that generate thousands of different mutants with unknown binding properties [38–40]. This makes using commonly used approaches, which include the purification of proteins for their detailed characterization, nearly impossible. The addition of a purification tag can simplify the task [41–43], although it can also cause a significantly decreased mutant production. As lectins lack any catalytic activity, it is not possible to use simple colorimetric assays as with enzymes [44, 45], where a crude cytoplasmic extract can be mixed with a chromogenic substrate and the colour change is detected.

Therefore, we decided to test and validate the applicability of simple agglutination techniques to differentiate subtle changes in lectin specificities.

### Optimisation of agglutination assay using CV-IIL

Human red blood cells (blood group 0) and yeast cells (*Saccharomyces cerevisiae*) were used for hemagglutination and yeast agglutination assays. RBCs of blood group 0 were chosen because they have more exposed terminal l-fucose than RBCs of blood group A or B, which is therefore readily accessible for interactions [26]. On the other hand, the cell wall of *S*. *cerevisiae* is rich in mannoproteins which bear branched mannans [46].

Optimization of the conditions for the agglutination assay was performed using CV-IIL. As it only exhibits minor differences in affinity towards l-fucose and d-mannose, it served for optimizing both the hemagglutination assay (HA) and the yeast agglutination assay (YA).

Three different concentrations of RBC or yeast suspensions (5, 10 and 20%), three different volumes of the sample drop (5, 10 and 15 μl) and a serial dilution of CV-IIL (from 0.1 mM) were tested to examine the effect of cell suspension concentration and its volume on the agglutination observations (S1, S2 and S3 Figs). All the experiments were repeated three times.

With YA, the **sample volume** played only a negligible role. In contrast, the sample volume had a significant effect on observations during HA experiments. Samples with a volume of 5 μl had a strong tendency to dry, which substantially affected the results. It was impossible to distinguish between a formation of small agglutinates and cell clumping caused by sample drying when low concentrations of CV-IIL were used for HA.

In both assays, high **concentrations of CV-IIL** gave large agglutinates clearly visible even with the naked eye on the microscope glass slide. However, high concentrations of CV-IIL in combination with a low amount of the yeast cells gave less stable agglutinates. The yeast agglutinates were disrupted when transferred onto the glass slide and covered with the cover glass in contrast to the RBC agglutinates, which were always very stable. On the other hand, a high amount of cells in combination with a low concentration of CV-IIL gave dubious results, as the very dense cell mass masked small agglutinates and made them poorly visible. The best results were obtained with a low amount of cells and a lower concentration of CV-IIL to enable a clearer identification of agglutinates.

In addition, two incubation times, 5 and 10 minutes, were tested in the YA assay but no significant differences between the results were observed.

For subsequent work, 5% cell suspensions, 10 μl sample volume, and 5 and 10 min of incubation were selected for all HA and YA assays, respectively.

### Optimisation of agglutination inhibition assays using CV-IIL

Serially diluted CV-IIL was used to find an appropriate concentration for subsequent hemagglutination inhibition (HI) and yeast agglutination inhibition (YAI) assays (S4 Fig). The concentration which caused a “total agglutination” (where all cells were cross-linked) and three lower concentrations were selected for subsequent agglutination inhibition assays. Inhibition assays were done with CV-IIL at concentrations of 400 nM, 200 nM, 100 nM, 50 nM for HI and 25 μM, 12.5 μM, 6.25 μM, 3.13 μM for YAI and were repeated three times. Four different concentrations were selected to differentiate the sensitivity and effectiveness of observing agglutinates of a different size, and to detect potential differences in the inhibitory potencies of the tested sugars.

Subsequently, serially diluted l-fucose and d-mannose were used as inhibitors to determine their ability to inhibit cell agglutination.

The minimal inhibitory concentration (MIC) which was necessary for agglutination inhibition, was determined for both sugars. To evaluate the results, the inhibition ratio was calculated, which was defined as the ratio of the MIC of d-mannose to that of l-fucose.

**Fig 3:**
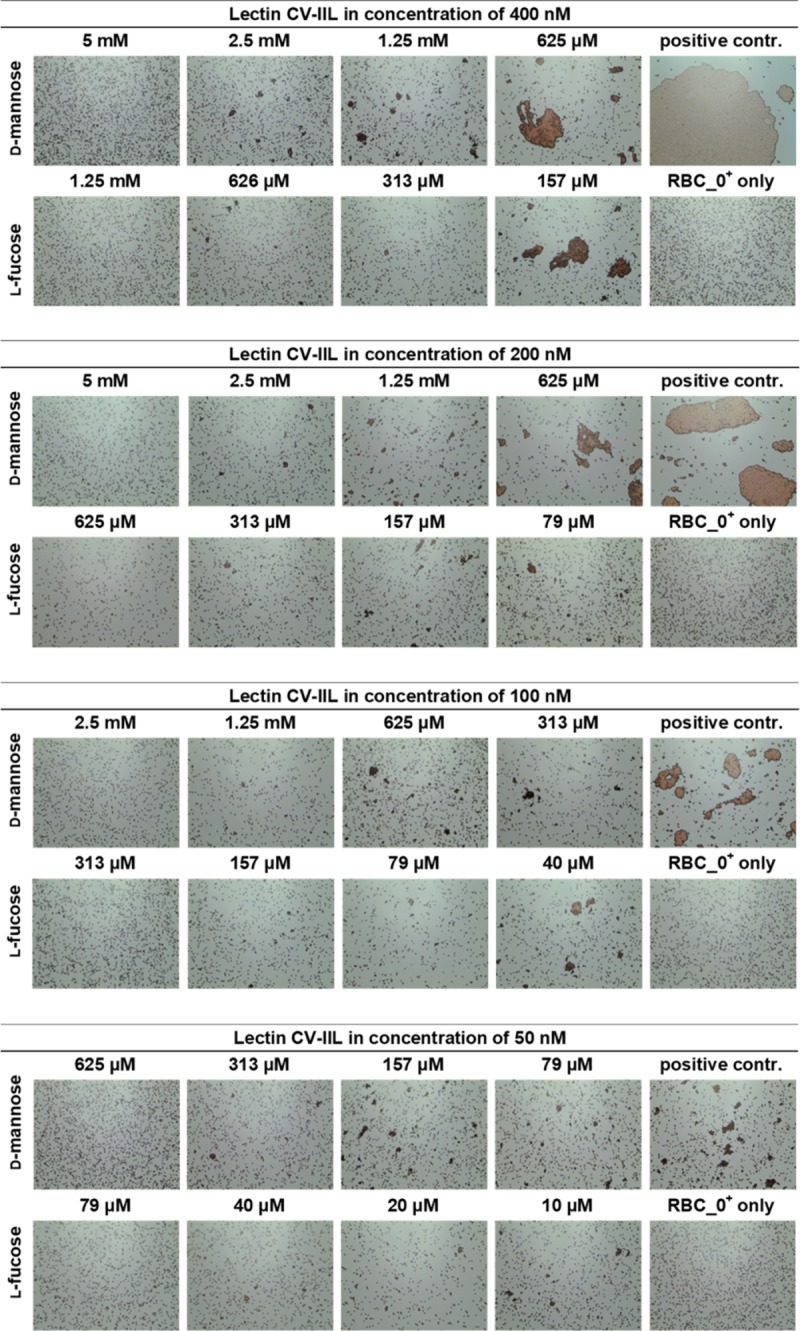
Hemagglutination inhibition assays with CV-IIL at final concentrations of 400 nM, 200 nM, 100 nM, and 50 nM. Each lectin sample was mixed with the monosaccharide in a 1: 1 ratio. The mixture was incubated for 1 minute at room temperature and 10 μl of 5% RBC_0^+^ was added immediately to the mixture. The mixture was incubated for an additional 5 minutes at room temperature, mixed again, applied to a glass slide and observed under a Levenhuk microscope. Pictures were taken with a DEM135 camera (Levenhuk). All negative control experiments did not exhibit any visible agglutination.

**Fig 4:**
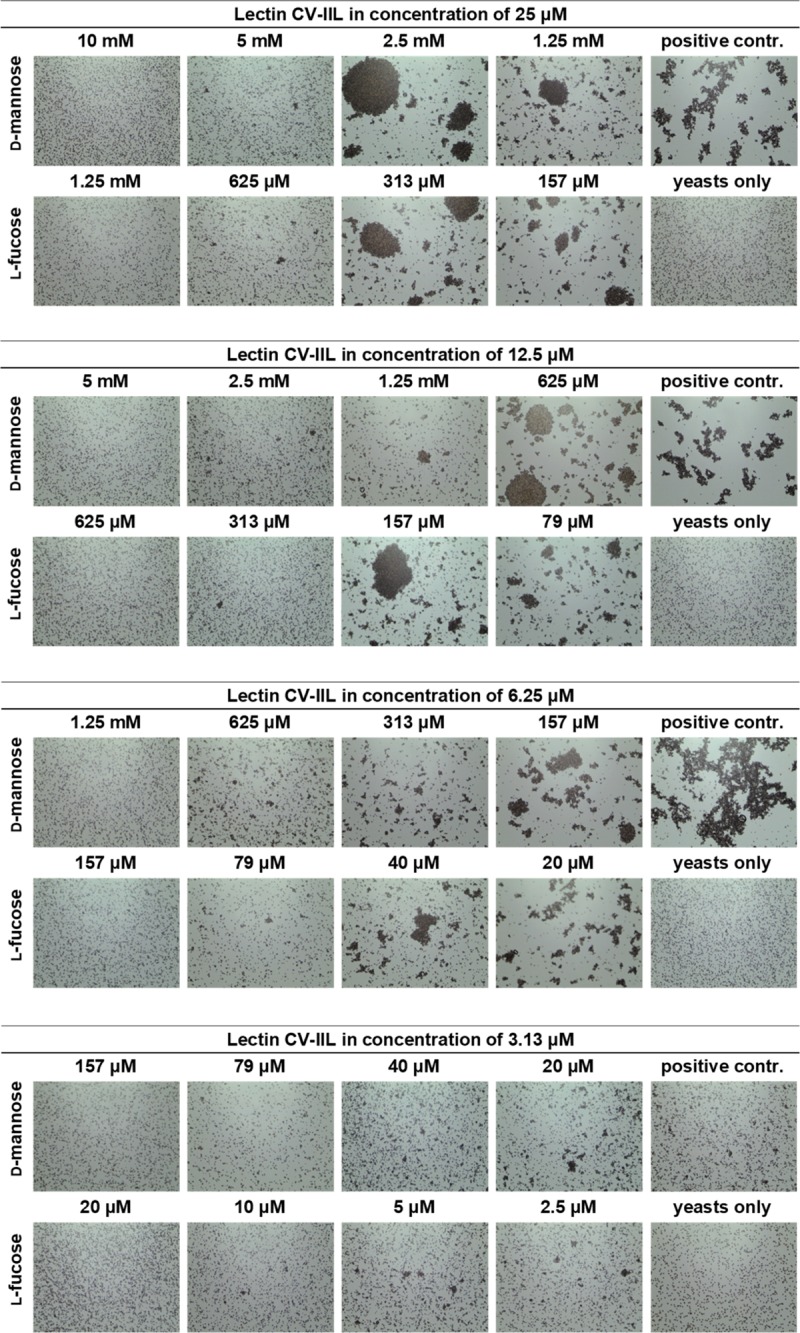
Yeast agglutination inhibition assays with CV-IIL at final concentrations of 25 μM, 12.5 μM, 6.25 μM, and 3.13 μM. Each lectin sample was mixed with the monosaccharide in a 1: 1 ratio. The mixture was incubated for 1 minute at room temperature, and 10 μl of 5% yeast cell suspension was added immediately to the mixture. The mixture was incubated for 10 additional minutes at room temperature, mixed again, applied to a glass slide and observed under a Levenhuk microscope. Pictures were taken with a DEM135 camera (Levenhuk). All negative control experiments did not exhibit any visible agglutination.

Results from HI showed that the MIC for d-mannose was 8-fold higher than for l-fucose using CV-IIL at concentrations of 200 nM, 100 nM, and 50 nM. However, there was a poorer detectability of agglutinates when using the two lowest concentrations. The inhibition ratio between d-mannose and l-fucose decreased to 4 when using CV-IIL at the concentration of 400 nM (Fig 3). This fact may suggest that the assay may lose its sensitivity using proteins at concentrations causing total hemagglutination. In the YAI assays, the inhibition ratio remained at 8 independently of the CV-IIL concentration (Fig 4) and poor distinguishability of agglutinates was only seen with CV-IIL at the lowest concentration.

The results of CV-IIL inhibition assays showed that this method was able to distinguish the subtle difference in lectin specificity. The inhibition ratio obtained by RBC and yeast agglutination inhibition methods were close to the ratio of binding affinities determined by ELLA and ITC measurements [36], enabling a semi-quantification of lectin affinity. Inhibition experiments further showed the necessity of always comparing the inhibitory potency of the inhibiting molecules with a standard (in our case the inhibition ratio of the two sugars) rather than to just evaluate their concentration necessary for inhibition. These concentrations are dependent on the arrangement of the experiment and can vary significantly. For example, the MIC for fucose varied from 0.3–2.5 mM and 0.04–0.6 mM for HI and YAI, respectively, while the inhibition ratios towards mannose remained stable. Similarly, determining the lectin concentration used for inhibition experiments is always necessary, as the lectin activity may vary with a particular lectin batch or over time. The variability of RBC or yeast cell batches must be also taken into account.

### Agglutination inhibition assays of RS-IIL_A22S

Based on the agglutination inhibition results obtained for CV-IIL, RS-IIL_A22S concentrations of 200 nM were selected for HI and 6.25 μM (corresponding to total agglutination) for YAI (S5 Fig). Neither HI nor YAI was repeated due to a low yield of purified RS-IIL_A22S caused by a low expression level of this mutant.

**Fig 5:**
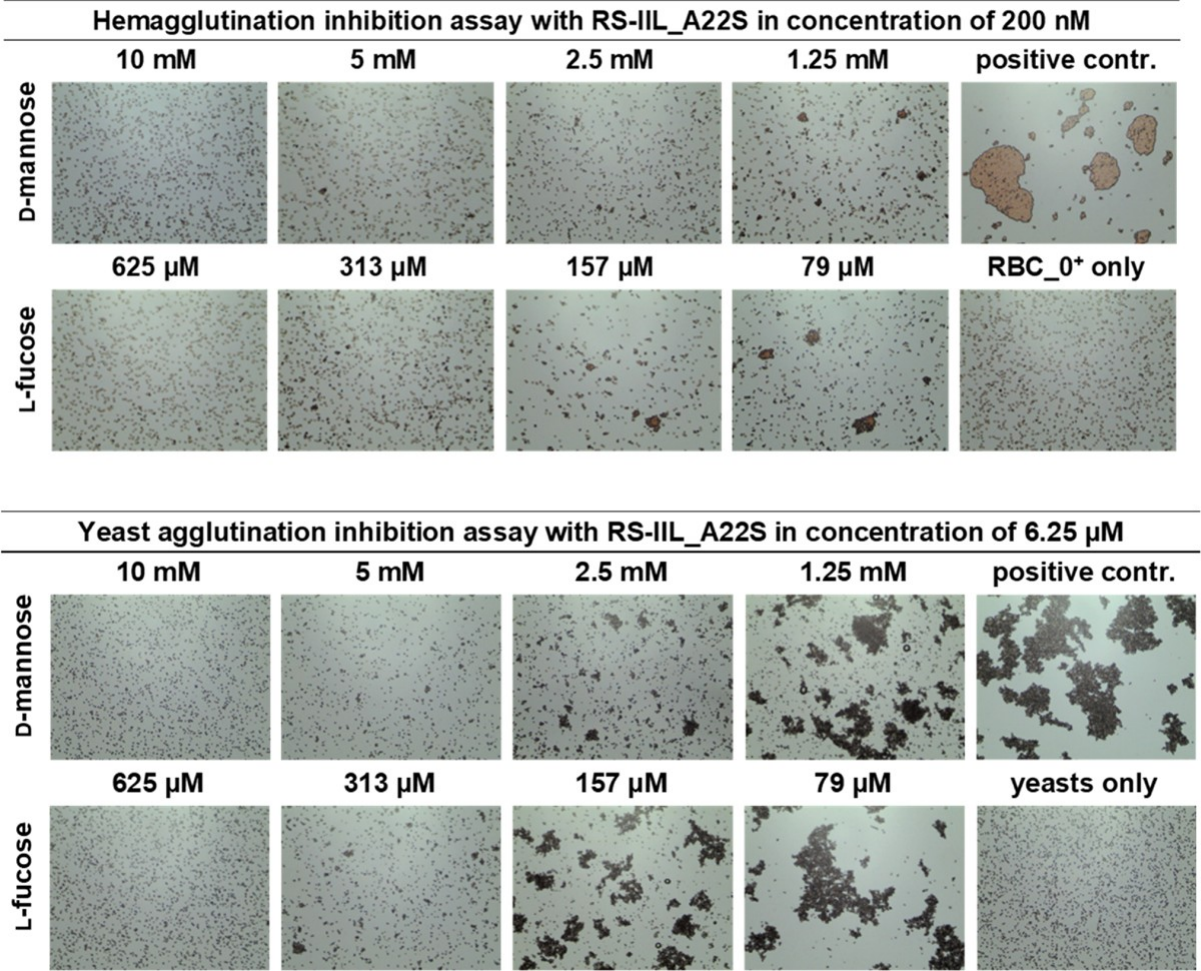
Hemagglutination (upper panel) and yeast agglutination inhibition assays (lower panel) with RS-IIL_A22S at final concentrations of 200 nM and 6.25 μM. Each lectin sample was mixed with the monosaccharide in a 1: 1 ratio. The mixture was incubated for 1 minute at room temperature, and then 10 μl of 5% RBC_0^+^ or yeast suspension was added to the mixture. The mixture was incubated at room temperature for an additional 5 minutes or 10 minutes, respectively, mixed again, applied to a glass slide and observed under a Levenhuk microscope. Pictures were taken with a DEM135 camera (Levenhuk). All negative control experiments did not exhibit any visible agglutination.

The results of the inhibition assays showed that the concentration of d-mannose necessary for agglutination inhibition was 16-fold higher than the inhibitory concentration of l-fucose, and the inhibition ratio remained the same with both cell types (Fig 5). The results indicated that the inhibition ratio determined by this method is very close to the ratio of binding affinities obtained by ITC measurement done with the RS-IIL mutant.

### Agglutination inhibition assays using crude bacterial cytoplasmic extracts

**Fig 6:**
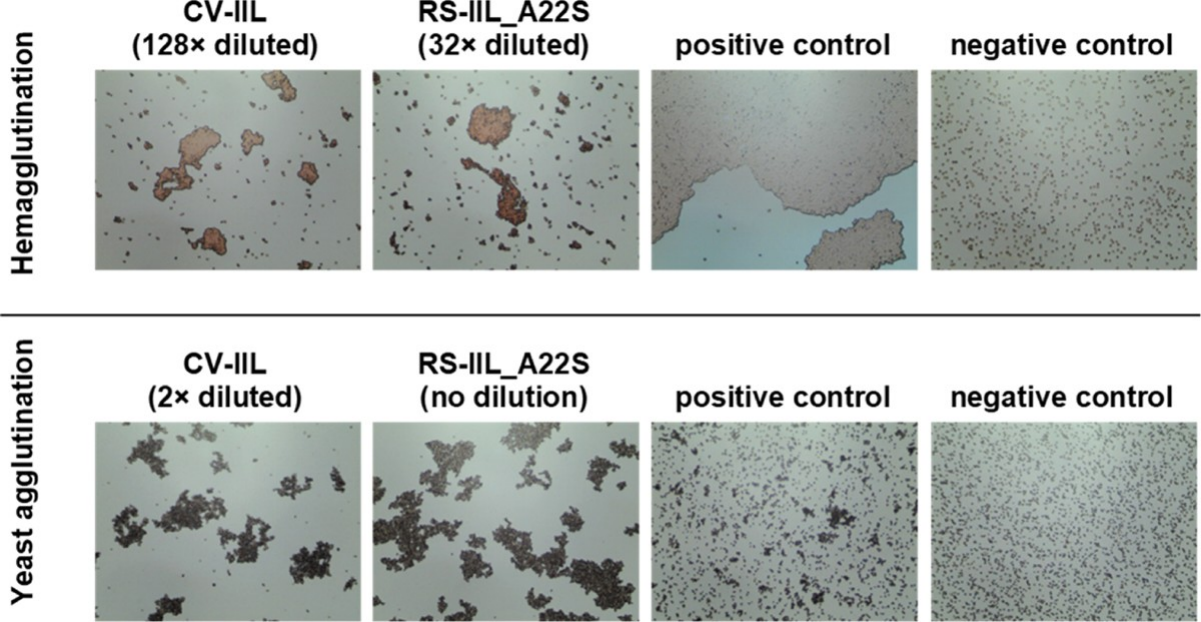
Determination of cytoplasmic extract dilution for hemagglutination and yeast agglutination inhibition assays. Cytosolic extract containing CV-IIL or RS-IIL_A22S was serially diluted in working buffer, and each sample from the dilution line was mixed with 5% RBC_0^+^ or yeast cell suspension in a 1: 1 ratio. The mixture was incubated at room temperature for 5 or 10 minutes, respectively, mixed again, applied to a glass slide and observed under a Levenhuk microscope. Pictures were taken with a DEM135 camera (Levenhuk). 128× diluted and 2× diluted cytoplasmic extract was chosen for the CV-IIL hemagglutination and yeast agglutination inhibition assay, respectively. 32× diluted and undiluted cytoplasmic extract were chosen for the RS-IIL_A22S hemagglutination and yeast agglutination inhibition assay, respectively. All negative control experiments did not exhibit any visible agglutination. The positive control was done using purified CV-IIL.

**Fig 7:**
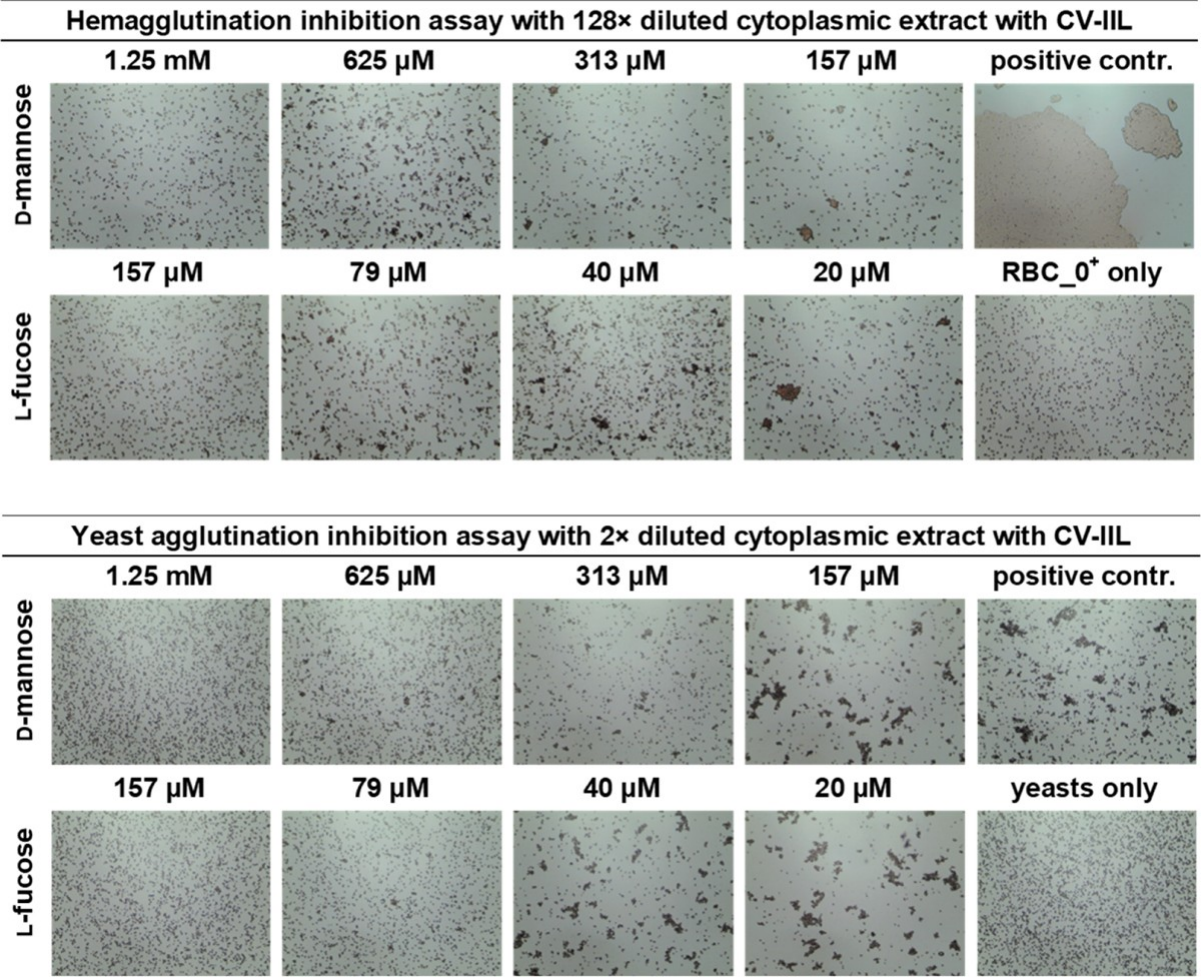
Agglutination inhibition assay to test specificity of CV-IIL directly in *E*. *coli* cytosol. Hemagglutination (upper panel) and yeast agglutination inhibition assays (lower panel) with 128× and 2× diluted cytoplasmic extract of *E*. *coli* expressing *cv-2l* gene, respectively. Diluted cytosol was mixed with the monosaccharide in a 1: 1 ratio and incubated for 1 minute at room temperature. 10 μl of the solution was mixed with 10 μl of 5% RBC_0^+^ or 5% yeast suspension and incubated at room temperature for an additional 5 (HI) or 10 (YAI) minutes, mixed again, applied to a glass slide and observed under a Levenhuk microscope. Pictures were taken with a DEM135 camera (Levenhuk).

**Fig 8:**
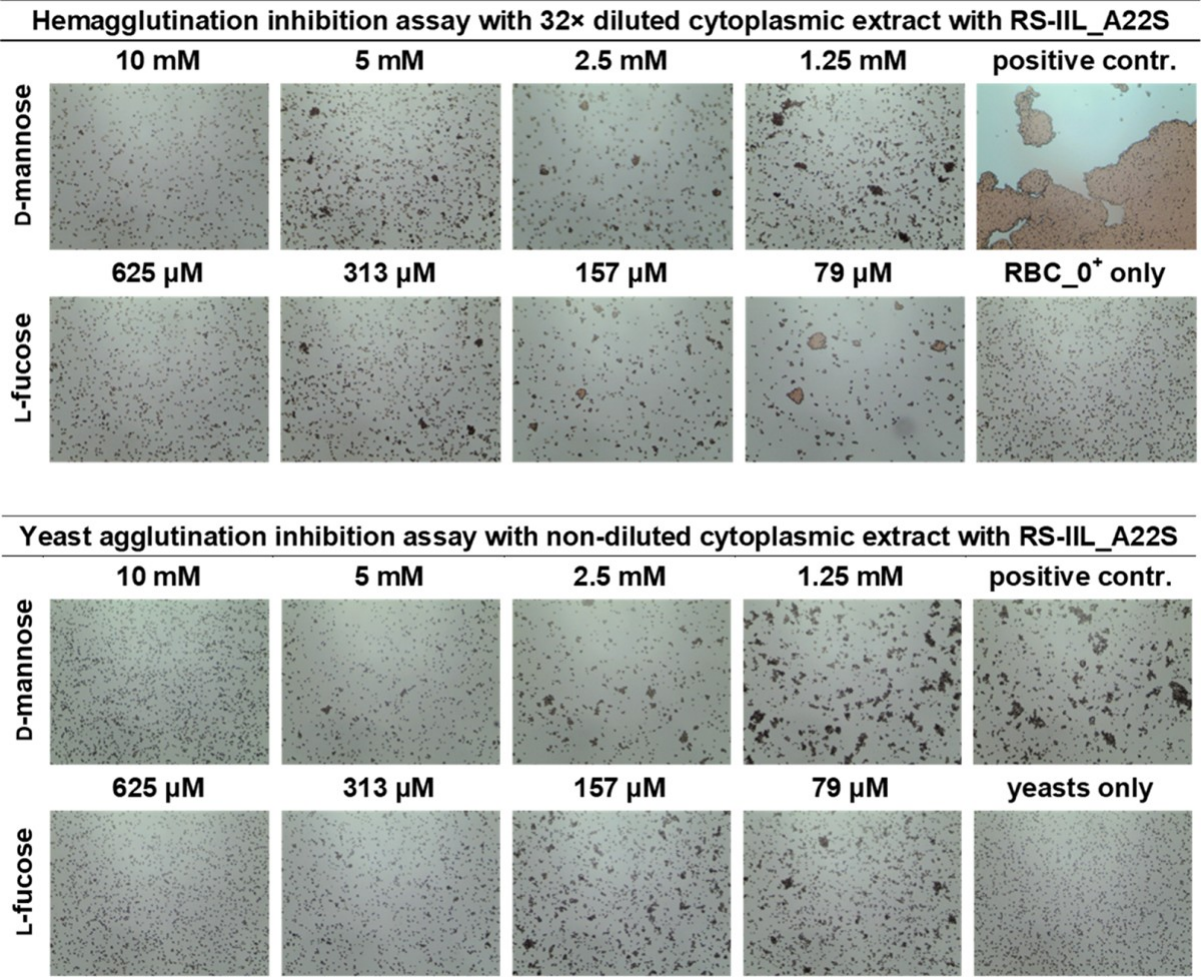
Agglutination inhibition assay to test specificity of RS-IIL_A22S directly in *E*. *coli* cytosol. Hemagglutination (upper panel) and yeast agglutination inhibition assays (lower panel) with 32× and undiluted cytoplasmic extract of *E*. *coli* expressing *rs-2l_A22S* gene, respectively. Diluted cytosol was mixed with the monosaccharide in a 1: 1 ratio and incubated for 1 minute at room temperature. 10 μl of the solution was mixed with 10 μl of 5% RBC_0^+^ or 5% yeast suspension and incubated at room temperature for an additional 5 (HI) or 10 (YAI) minutes, mixed again, applied to a glass slide and observed under a Levenhuk microscope. Pictures were taken with a DEM135 camera (Levenhuk).

To determine if those small differences in lectin specificities can be directly seen without needing protein purification, several experiments using *E*. *coli* cell lysates after the induction of protein production were performed. Hemagglutination and yeast agglutination assays were done with serially diluted cytoplasmic extracts containing CV-IIL and RS-IIL_A22S, respectively, to determine the appropriate dilution for subsequent inhibition assays (S6 and S7 Figs). For HI, a dilution that did not cause total agglutination was chosen (128-fold and 32-fold dilution for CV-IIL and RS-IIL_A22S, respectively, Fig 6). On the other hand, the dilution causing total agglutination was used for YAI for the best visibility (2-fold and undiluted lysate for CV-IIL and RS-IIL_A22S, respectively). *E*. *coli* Tuner (DE3) lacking a plasmid was used as a negative control, and no agglutination was seen at any dilution tested (S10 Fig). The results showed that both agglutination inhibition assays performed directly with the bacterial extracts gave the same results as experiments with purified lectins, i.e., the inhibition ratio for mannose/fucose was 8 with CV-IIL (Fig 7) and 16 with the mutant RS-IIL_A22S (Fig 8). Experiments with wtRS-IIL in a crude bacterial cytoplasmic extract were also performed. However, we were not able to reach total agglutination in agglutination assays, which was probably caused by a low expression level of wtRS-IIL (S8 Fig). Nevertheless, agglutination inhibition assays were performed. The HI results showed that d-mannose is 256-fold better inhibitor than l-fucose. On the other hand, results of YAI were inconclusive (S9 Fig).

### Hemagglutination inhibition assays on microtiter plate

Hemagglutination assay with microscopy detection was validated by a comparison with standard HI assay on the microtiter plate. Proteins CV-IIL and RS-IIL_A22S and bacterial extracts were used for this assay. Three concentrations of RBC suspension (1%, 2% and 4%) were tested using CV-IIL. Subsequently, 2% RBC suspension was chosen for all standard HI assays because it enabled the best observation of agglutination in the U-bottomed 96-well microtiter plate.

**Fig 9:**
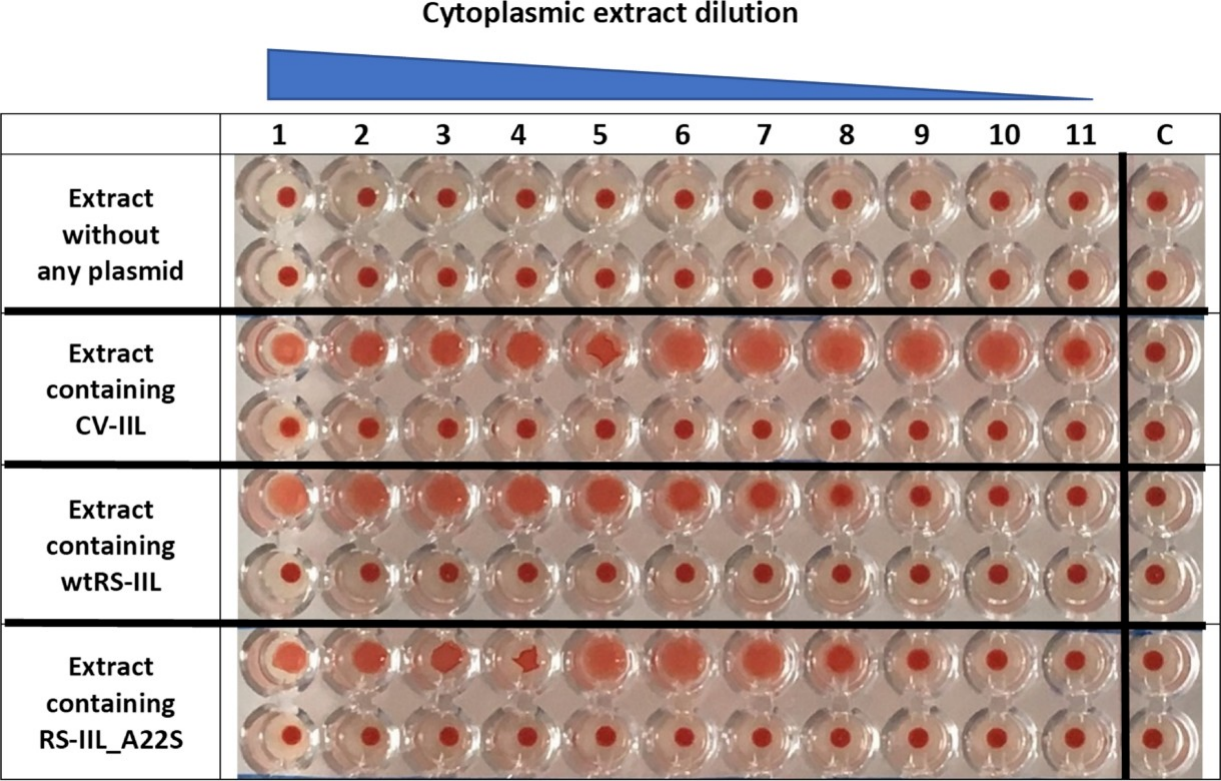
Determination of cytoplasmic extract titer for hemagglutination inhibition assay on microtiter plate. Bacterial cytoplasmic extracts (undiluted in the first well) dilution increases from left to right in two rows by a ratio of 0.5 between two neighboring wells. Agglutinated red blood cells form a diffuse mat, whereas non-agglutinated red blood cells sediment and form a clear dot in the bottom of the well. The titer was determined for extract containing CV-IIL (well 10 in the first row), wtRS-IIL (well 5 in the first row) and RS-IIL_A22S (well 7 in the first row). *E*. *coli* cytoplasmic extract without any plasmid served as negative control. Last wells represent control experiments in absence of lectin.

**Fig 10:**
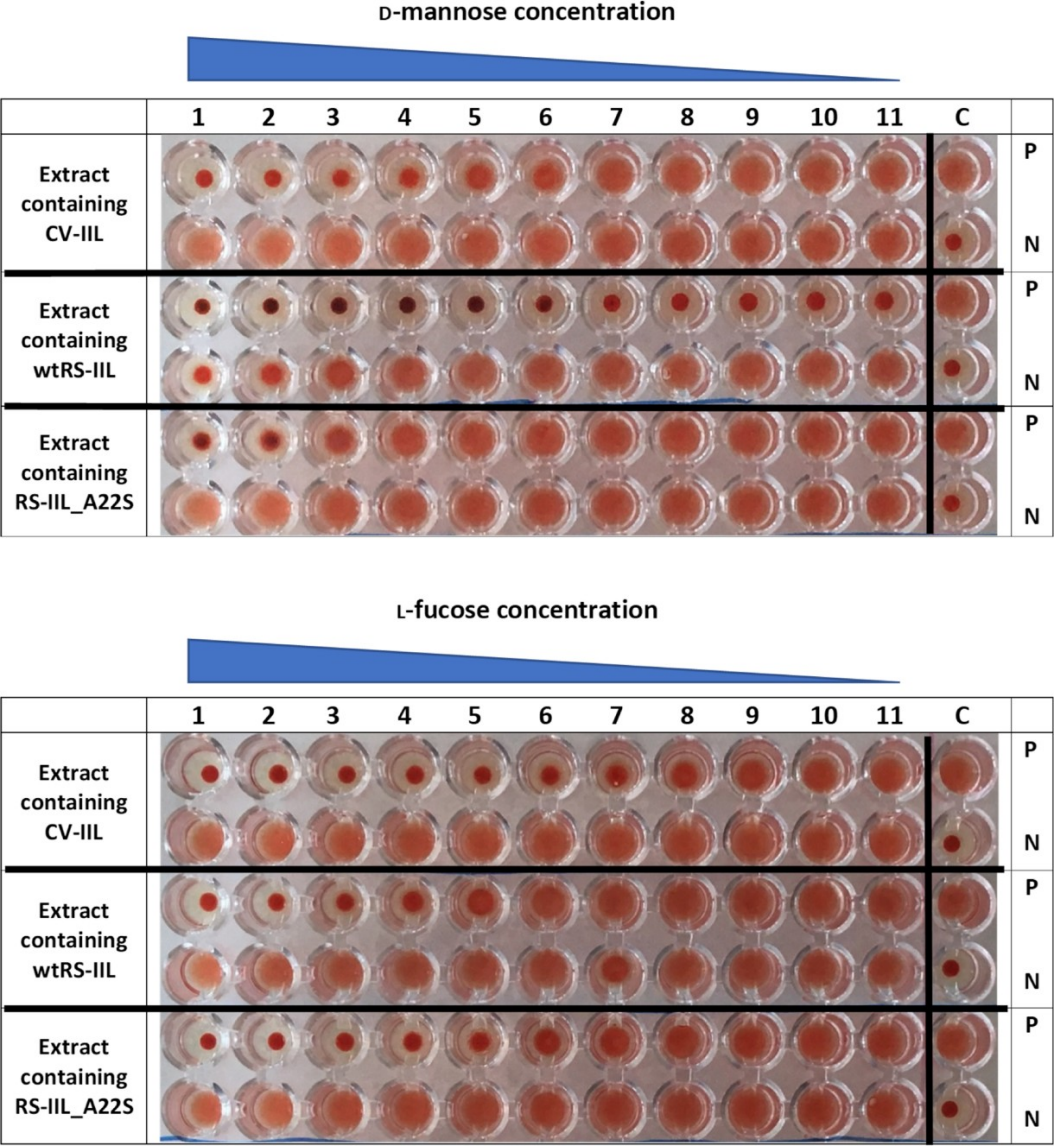
Hemagglutination inhibition assay on microtiter plate of cytoplasmic extract containing lectins. Cytoplasmic extracts containing lectins in dilution corresponding to titer 1 were used. The first well in the first row contains 40 mM d-mannose (upper panel) or l-fucose (lower panel). 8 mM d-mannose or l-fucose were used for cytoplasmic extract containing RS-IIL_A22S. Carbohydrate concentration decreases from left to right in two rows by a ratio of 0.5 between two neighboring wells. Inhibited agglutination results in a clear dot in the bottom of the well, whereas non-inhibited agglutination results in a diffuse mat. Last wells represent control experiments. In positive controls (P) buffer was used instead of monosaccharide. In a negative controls (N) buffer was used instead of lectin.

CV-IIL titer (the highest dilution of the lectin solution showing complete agglutination) was determined (S11 Fig). The concentration of CV-IIL titer for 2% RBC suspension was 100 nM. HI assays on microtiter plate with four concentrations of CV-IIL corresponding to titers 0.5, 1, 2 and 4 were performed. The results were the same for all CV-IIL concentrations used, l-fucose was 8-fold better inhibitor than d-mannose (S12 Fig). Using RS-IIL_A22S, HI assays only with titer 1 were performed. Fucose concentration necessary for inhibition of RS-IIL_A22S agglutination was 16-fold lower than the inhibitory concentration of mannose (S13 Fig). In case of bacterial extracts (titer in Fig 9) HI assays showed the mannose/ fucose inhibition ratio was 4 or 8 for extract containing CV-IIL or RS-IIL_A22S, respectively. The results slightly differed from results obtained from HI assays with cytoplasmic extract examined by microscopy where the inhibition ratio was 8 or 16 for CV-IIL or RS-IIL_A22S, respectively. In the case of extract containing wtRS-IIL, d-mannose was 512-fold better inhibitor than l-fucose. This result again differed from HI assay examined by microscopy where the d-mannose was 256-fold better inhibitor. In both cases the difference was one dilution (Fig 10). Control experiments showed no agglutination using the *E*. *coli* Tuner (DE3) extracts without any plasmid (Fig 9).

The inhibition ratios between l-fucose and d-mannose obtained by HI assays on microtiter plate with purified lectins were identical and with cytoplasmic extracts slightly different compared to results obtained by HI assays examined by microscopy. Altogether with ITC and SPR results it confirms the validity of the method.

However, in some cases and particularly with cytoplasmic extracts, it was difficult to clearly distinguish complete inhibition from partial inhibition of agglutination in HI assays on the microtiter plate and therefore to exactly determine MIC. It is a common problem because HI assays on the microtiter plate are usually evaluated visually by the naked eye and therefore determination of results depends on the examiner experience. Zhao et al. were not able to clearly distinguish samples that were partially-agglutinated from the samples with completely inhibited agglutination during HI assays evaluating inhibitory activities of pectin-derived polysaccharides to galectin-3. Therefore, they developed a digital data detection method for HI assay using microplate reader [47].

Lectin-based agglutination assays of cells or artificial particles tend to be coupled with methods that enable more accurate determination of results and eliminate factor of subjectivity in the process of evaluation. Quantification of non-agglutinated cells remaining in suspension by an electronic cell counter was used for evaluation of hemagglutination assay of phytohemagglutinin from kidney beans [48]. Flemming et al. designed a simple apparatus called agglutimeter which utilized a photometer for measurement of turbidity of samples containing agglutinates. This technique can be applied not only to agglutination of erythrocytes but all types of cells [49]. Optical density, turbidity or dynamic light scattering were measured when agglutination assays with glycan-decorated vesicles was used for investigation of lectin multivalent interactions [50] or lectin hetero-multivalency [51]. These methods were also utilized to study lectin-mediated aggregation of glycosylated nanoparticles [52].

All these techniques provided consistent, objective and precise results, however additional instruments, which may not be present at all laboratories, are needed. On the other hand, the agglutination inhibition assay examined by microscopy enables a quick and non-expensive solution to this problem because it enables distinguishing of even small agglutinates using a small portable bright-field microscope, which was used in this study.

### Influence of lectins on RBC fitness

Hemagglutination assays on microtiter plate is not applicable if lectins are cytotoxic or show hemolytic activity as hemolysis strongly affects the evaluation of agglutination results. Furthermore, binding of multivalent lectins can have an impact on a shape of membranes [53], even though they are not classified as cytotoxic. In addition, RBC used in agglutination experiments with lectins are often treated by papain and this treatment has a significant effect on integrity of the cell membrane [54]. The long-time incubation needed for evaluation of the HI assay on microtiter plates can significantly increase possible negative effects of lectins on RBCs.

Neither CV-IIL nor RS-IIL_A22S are cytotoxic and the results of HI assay on microtiter plate with these lectins were recognisable. Nevertheless, the effect of the lectins on red blood cell condition was tested. Serial dilution of CV-IIL or RS-IIL_A22S was done and after 1 hour of incubation with RBC the sample corresponding to titer 1 was examined under the microscope. The deformed shape of cells forming agglutinates was clearly observable (S14 Fig). The experiment showed that even proteins which have not been classified as cytotoxic or hemolytic may affect the cell fitness in the HI assay on the microtiter plate even if they are used in a low concentration.

Therefore, the method of the agglutination examined by microscopy is particularly suitable for examination of cytotoxic lectins and lectins with hemolytic activity as well as of unknown lectins or lectin mutant variants, which could have unpredictable effects on RBC condition. In contrast to hemagglutination assay on microtiter plate, the experimental arrangement of this method enables a very short incubation time and precise distinguishing of hemagglutinates from hemolysis.

## Conclusion

The mutagenesis approach is frequently employed for detailed investigations of lectin properties, particularly its specificity. The characterization of lectin properties is a complex process that involves a spectrum of methods, most of which require sophisticated and expensive instrumentation. This process is often complicated by problematic behaviour of the studied lectins such as poor solubility or low stability under the required conditions.

Therefore, we searched for a fast and simple method that enables the evaluation of changes in lectin binding properties without requiring their purification, and agglutination inhibition assay with microscopy detection was successfully employed for this purpose. This method is simple, robust and non-expensive in the arrangement using RBCs or baker’s yeast and a small portable microscope. The sensitivity of this assay was tested with the fucose/mannose specific lectin CV-IIL with the saccharides l-fucose and d-mannose, and the inhibition ratio between these two sugars was similar to the ratio determined by ELLA and ITC methods. Also, the results obtained by the method were in concordance with the results from HI assays performed in standard format of a 96-well microtiter plate.

On the contrary to the assay on microtiter plate, cytotoxic proteins and proteins with hemolytic activity could be characterized by the agglutination assay with microscopy detection because experimental arrangement of the method enables short time of incubation which can reduce negative effects of the proteins on red blood cells.

In this paper, we also characterised a new mutant, RS-IIL_A22S, which confirmed the importance of the first amino acid in the specificity binding loop for sugar preference in the PA-IIL family. Using this to date uncharacterized mutant, we showed that outcomes from an amino acid substitution can be followed using simple AI on crude bacterial extracts. The results were similar to those obtained with the purified lectin using SPR and ITC. However, while the first strategy can be done in a few hours, work with purified proteins and their characterisation using more sophisticated methods can take days or weeks.

## Ethics statement

Anonymised human blood of blood groups A, B, O treated with natrium citrate was purchased from Transfusion and Tissue Department, The University Hospital Brno, Czech Republic. IRB approval for use of these samples is not requested.

## Supporting information

S1 Fig Optimization of RBC concentration used in the agglutination procedure and volume of mixture applied to a glass slide before observation under microscope. (PDF)

S2 Fig Optimization of yeast cell suspension concentration used in the agglutination procedure and volume of mixture applied to a glass slide before observation under microscope. (PDF)

S3 Fig Optimization of yeast cell suspension concentration used in the agglutination procedure and volume of mixture applied to a glass slide before observation under microscope. (PDF)

S4 Fig Determination of CV-IIL concentrations appropriate for agglutination inhibition assays. (PDF)

S5 Fig Determination of RS-IIL_A22S concentrations appropriate for agglutination inhibition assays. (PDF)

S6 Fig Determination of cytoplasmic extract dilution appropriate for hemagglutination inhibition assays. (PDF)

S7 Fig Determination of cytoplasmic extract dilution appropriate for yeast agglutination inhibition assays. (PDF)

S8 Fig Analysis of working cytoplasmic extract dilutions for inhibition assays. (PDF)

S9 Fig Agglutination inhibition assay to test specificity of wtRS-IIL directly in *E*. *coli* cytosol. (PDF)

S10 Fig Analysis of cytoplasmic extract of *E*. *coli* Tuner (DE3) lacking a plasmid. (PDF)

S11 Fig Determination of RBC suspension concentration and CV-IIL titers for HI assay on microtiter plate. (PDF)

S12 Fig Hemagglutination inhibition assay on microtiter plate with four concentrations of CV-IIL. (PDF)

S13 Fig Determination of RS-IIL-A22S titer and hemagglutination assay on microtiter plate. (PDF)

S14 Fig RBC condition after 1 hour of incubation with CV-IIL and RS-IIL_A22S in concentration of the titer. (PDF)
