# Supplementary material for: Microscopy examination of red blood and yeast cell agglutination induced by bacterial lectins
Source: PLoS One. 2019 Jul 25;14(7):e0220318. doi: 10.1371/journal.pone.0220318 (PMC6657890; doi:10.1371/journal.pone.0220318)
Supplement: S1 Fig — (PDF) [file pone.0220318.s001.pdf]

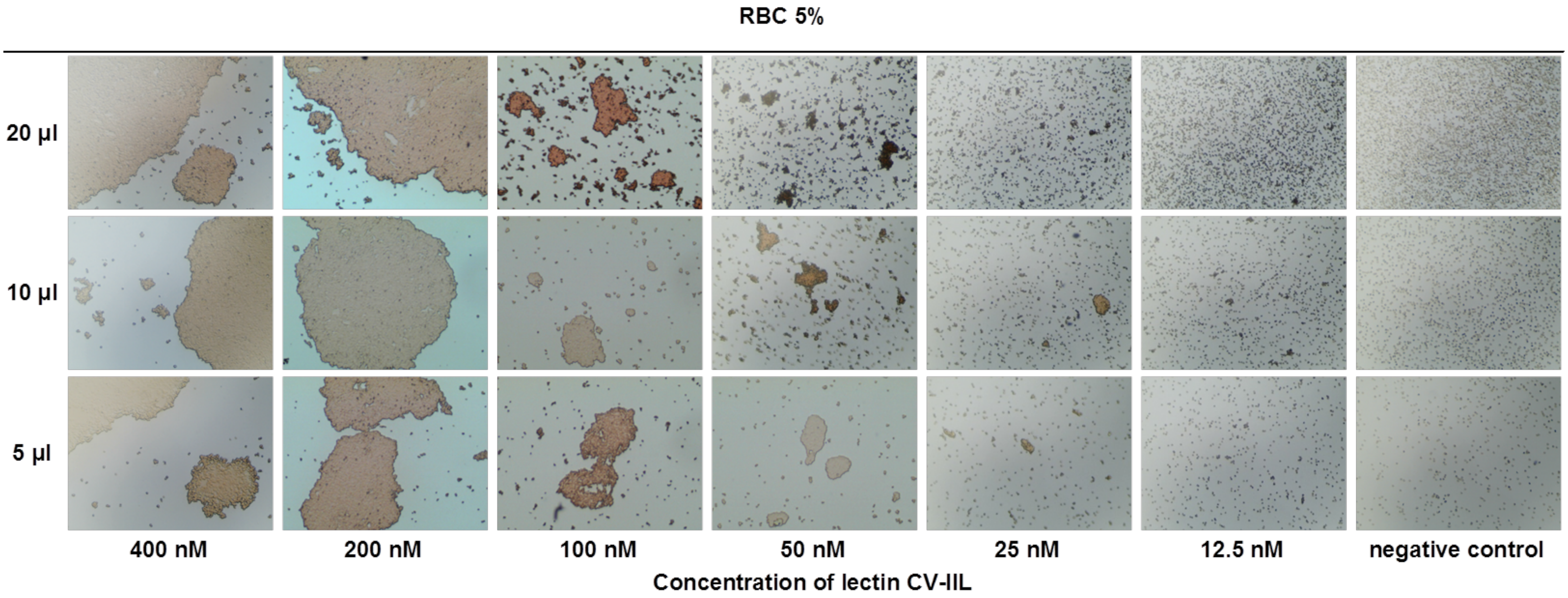
RBC 5%
20 µl
10 µl
5 µl
400 nM
200 nM
100 nM
50 nM
25 nM
12.5 nM
negative control
Concentration of lectin CV-IIL

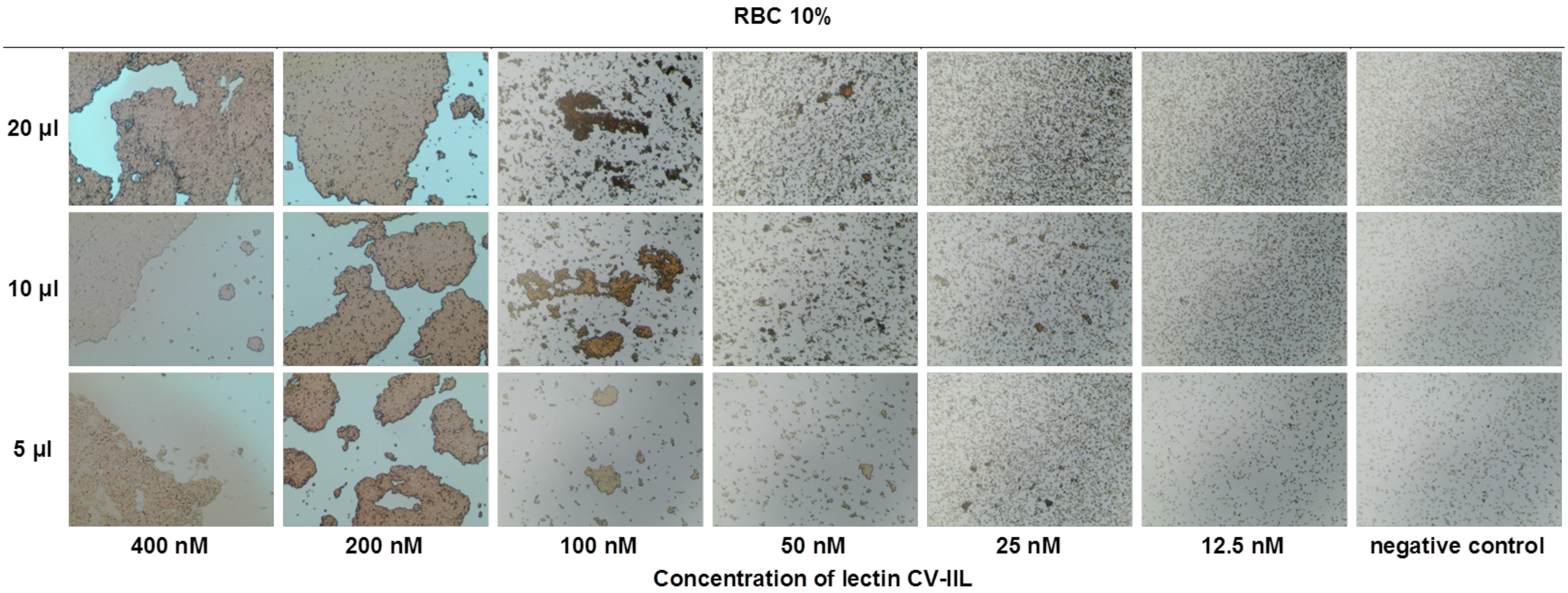

RBC 10%
20 µl
10 µl
5 µl
400 nM
200 nM
100 nM
50 nM
25 nM
12.5 nM
negative control
Concentration of lectin CV-IIL

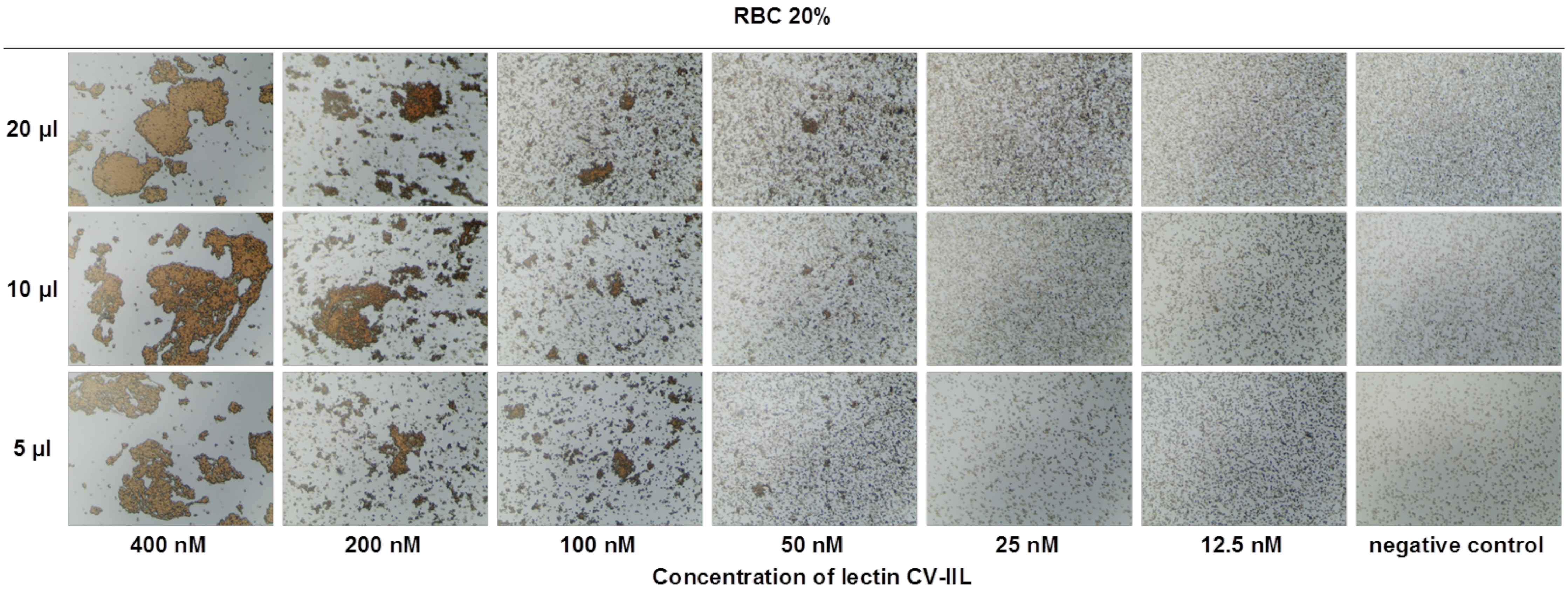

Fig. S1. Optimization of RBC concentration used in the agglutination procedure and volume of mixture applied to a glass slide before observation under microscope. Lectin CV-IIL was utilized in this optimization process. The lectin was serially diluted in working buffer and sample of each concentration was mixed in 1 : 1 ratio with suspensions of 5% RBC_0^{+} (upper panel) or 10% RBC_0^{+} (middle panel) or 20% RBC_0^{+} (lower panel). Mixture was incubated for 5 minutes at room temperature and mixed again. Three samples of each mixture in volumes 20 µl, 10 µl and 5 µl were applied to three glass slides and observed under the Levenhuk microscope. Pictures were taken by the camera DEM135 (Levenhuk). RBC hemolysis is apparent in mixtures of 5% RBC or 10% RBC with lectin CV-IIL in concentration 400 nM. All negative control experiments did not show any visible agglutination.
