# Supplementary material for: Microscopy examination of red blood and yeast cell agglutination induced by bacterial lectins
Source: PLoS One. 2019 Jul 25;14(7):e0220318. doi: 10.1371/journal.pone.0220318 (PMC6657890; doi:10.1371/journal.pone.0220318)
Supplement: S2 Fig — (PDF) [file pone.0220318.s002.pdf]

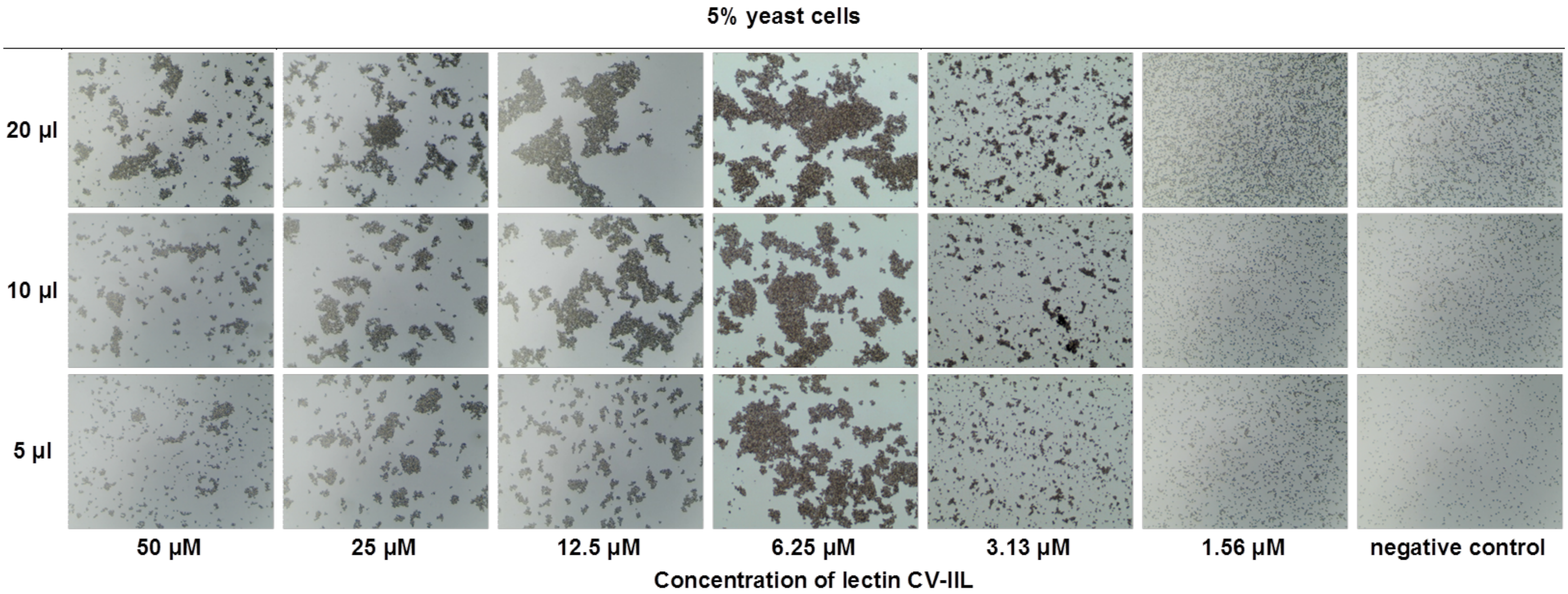
5% yeast cells
20 µl
10 µl
5 µl
50 µM
25 µM
12.5 µM
6.25 µM
3.13 µM
1.56 µM
negative control
Concentration of lectin CV-IIL

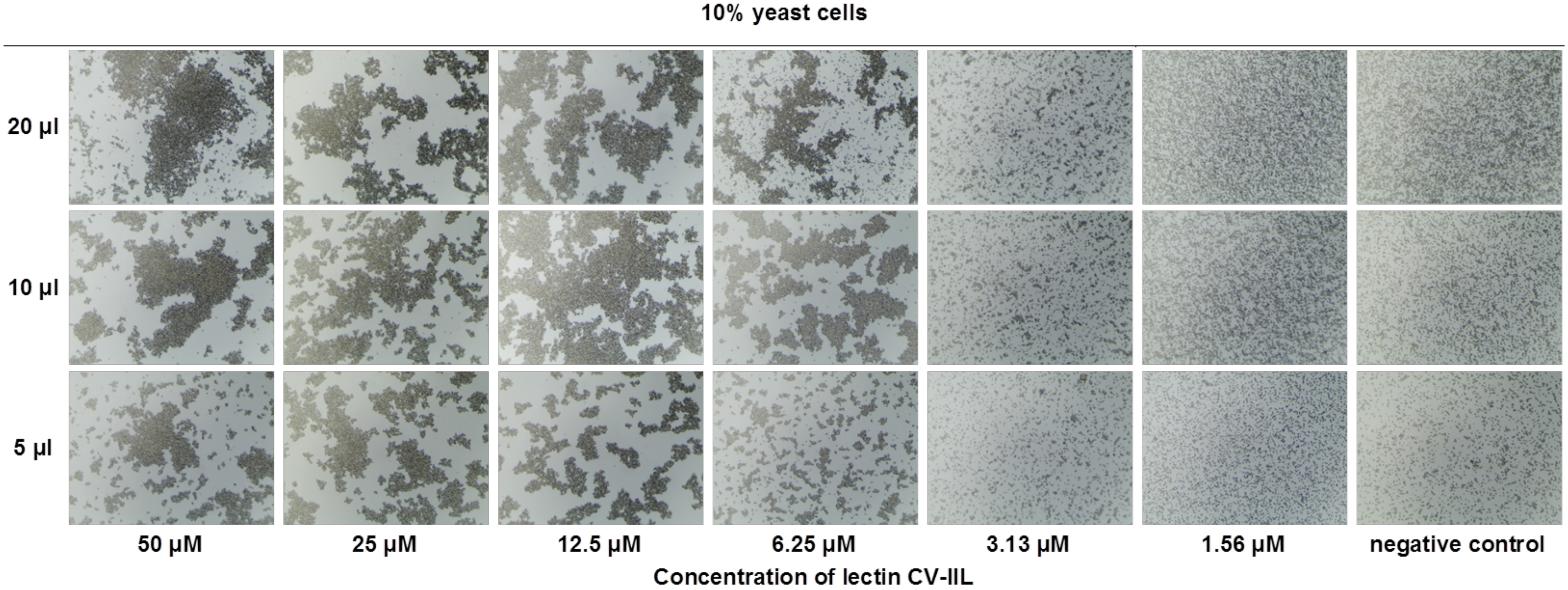
10% yeast cells
20 µl
10 µl
5 µl
50 µM
25 µM
12.5 µM
6.25 µM
3.13 µM
1.56 µM
negative control
Concentration of lectin CV-IIL

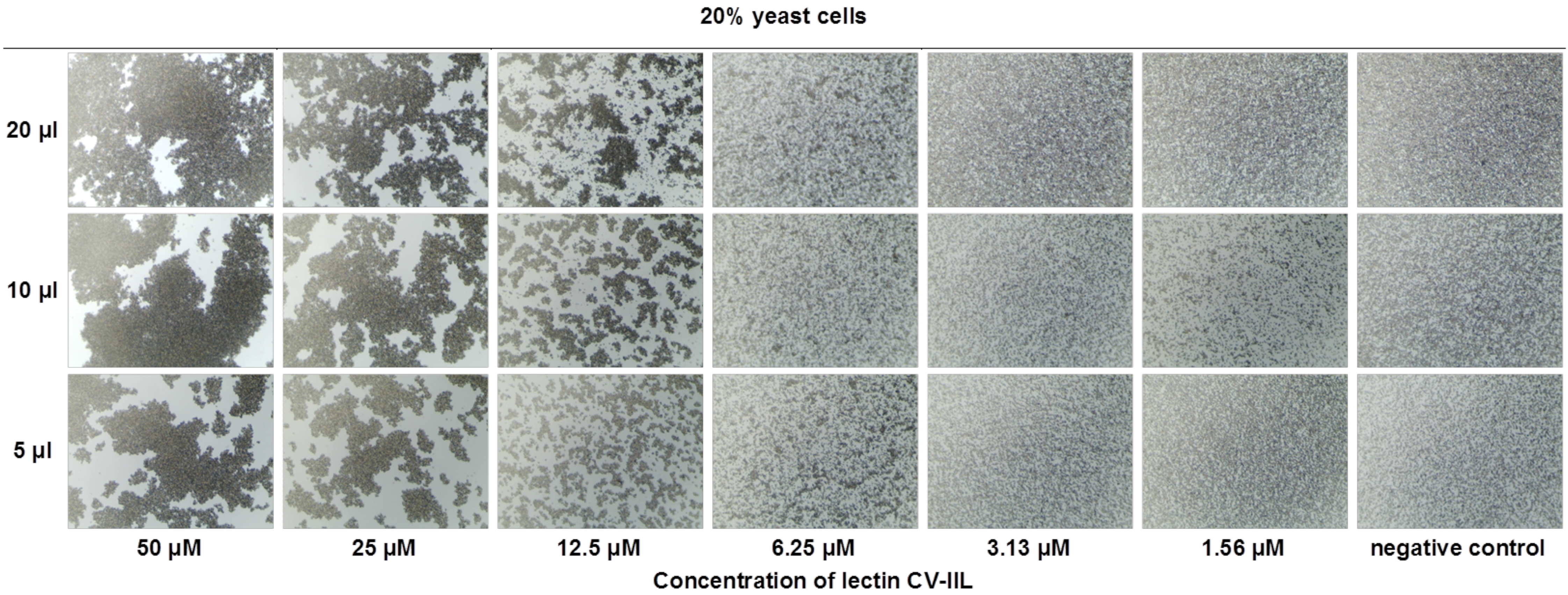

Fig. S2. Optimization of yeast cell suspension concentration used in the agglutination procedure and volume of mixture applied to a glass slide before observation under microscope. Lectin CV-IIL was utilized in this optimization process. The lectin was serially diluted in working buffer and sample of each concentration was mixed in 1 : 1 ratio with yeast cell suspensions of 5% (upper panel) or 10% (middle panel) or 20% (lower panel). Mixture was incubated for 5 minutes at room temperature and mixed again. Three samples of each mixture in volumes 20 µl, 10 µl and 5 µl were applied to three glass slides and observed under the Levenhuk microscope. Pictures were taken by the camera DEM135 (Levenhuk). Agglutinates made of 5% yeast cell suspension and higher CV-IIL concentrations are susceptible to damage when mixed again after incubation and transferred on the glass slide. All negative control experiments did not show any visible agglutination.
