# Supplementary material for: Microscopy examination of red blood and yeast cell agglutination induced by bacterial lectins
Source: PLoS One. 2019 Jul 25;14(7):e0220318. doi: 10.1371/journal.pone.0220318 (PMC6657890; doi:10.1371/journal.pone.0220318)
Supplement: S5 Fig — (PDF) [file pone.0220318.s005.pdf]

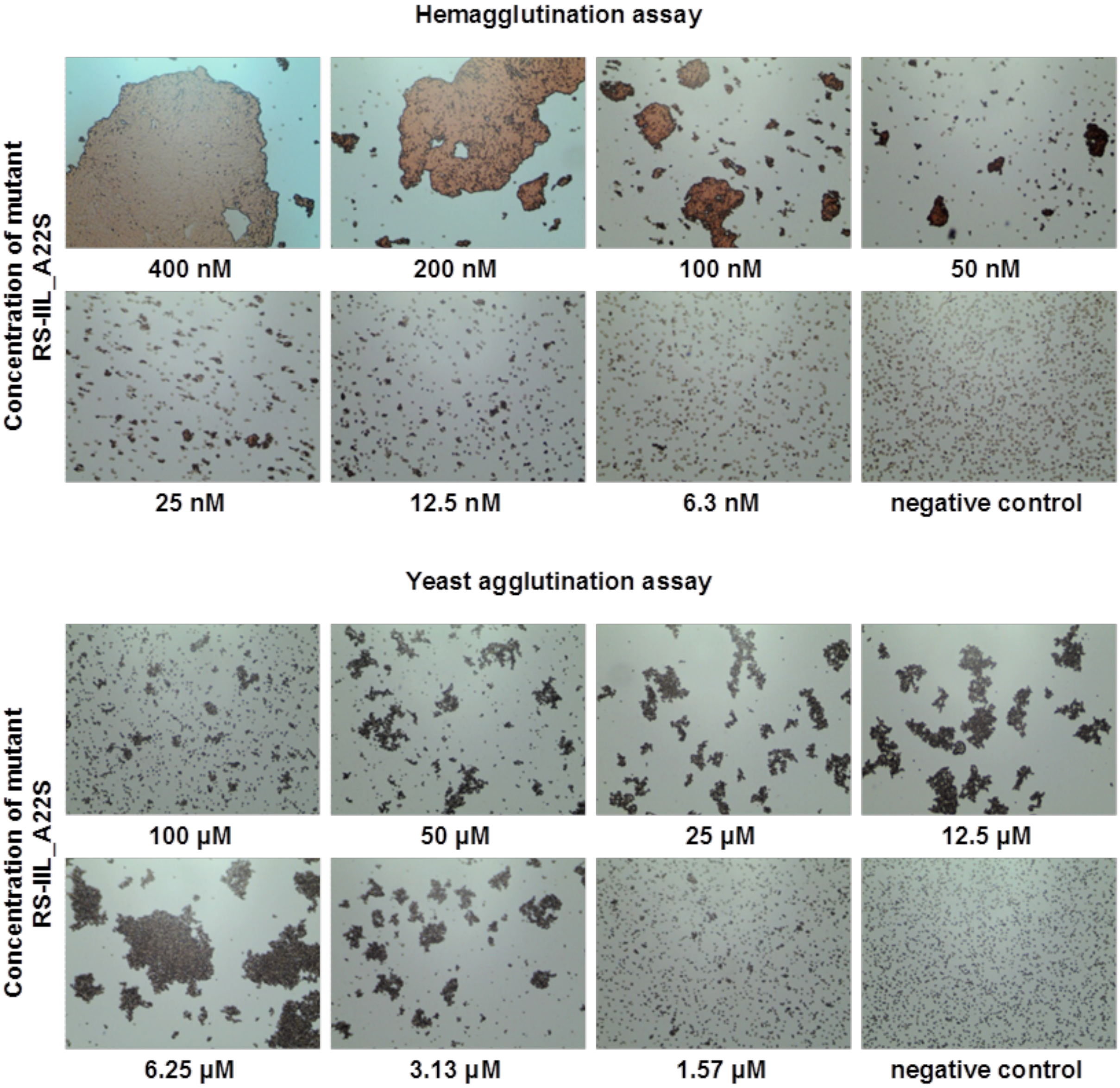

Fig. S5. Determination of RS-IIL_A22S concentrations appropriate for agglutination inhibition assays. Mutant RS-IIL_A22S was serially diluted in working buffer and sample of each concentration was mixed with 5% RBC_0^+ or yeast suspension in 1 : 1 ratio. Mixture was incubated at room temperature for 5 minutes or 10 minutes, respectively, mixed again, applied to a glass slide and observed under the Levenhuk microscope. Pictures were taken by the camera DEM135 (Levenhuk). RS-IIL_A22S in concentration of 200 nM was used in hemagglutination inhibition assay and its concentration of 6.25 µM was selected for yeast agglutination inhibition assay. All negative control experiments did not show any visible agglutination.
