# Supplementary material for: Microscopy examination of red blood and yeast cell agglutination induced by bacterial lectins
Source: PLoS One. 2019 Jul 25;14(7):e0220318. doi: 10.1371/journal.pone.0220318 (PMC6657890; doi:10.1371/journal.pone.0220318)
Supplement: S6 Fig — (PDF) [file pone.0220318.s006.pdf]

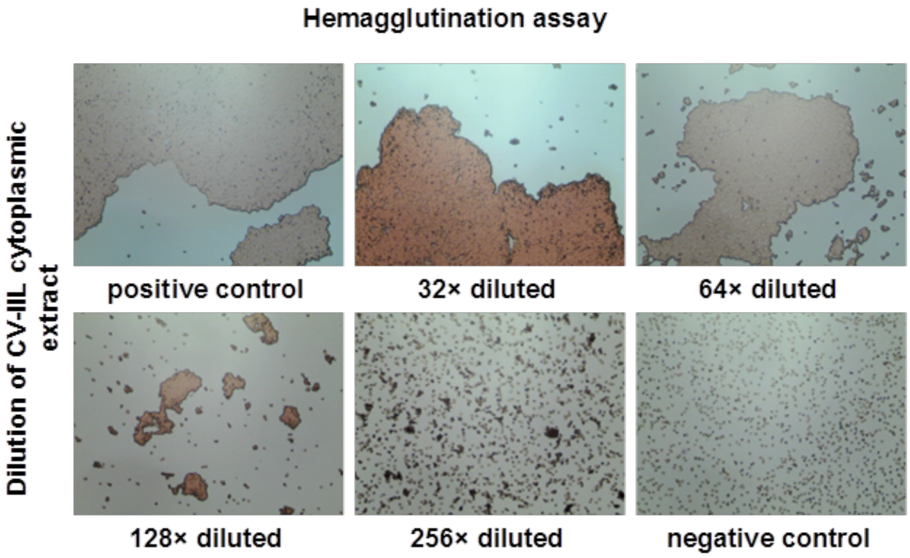

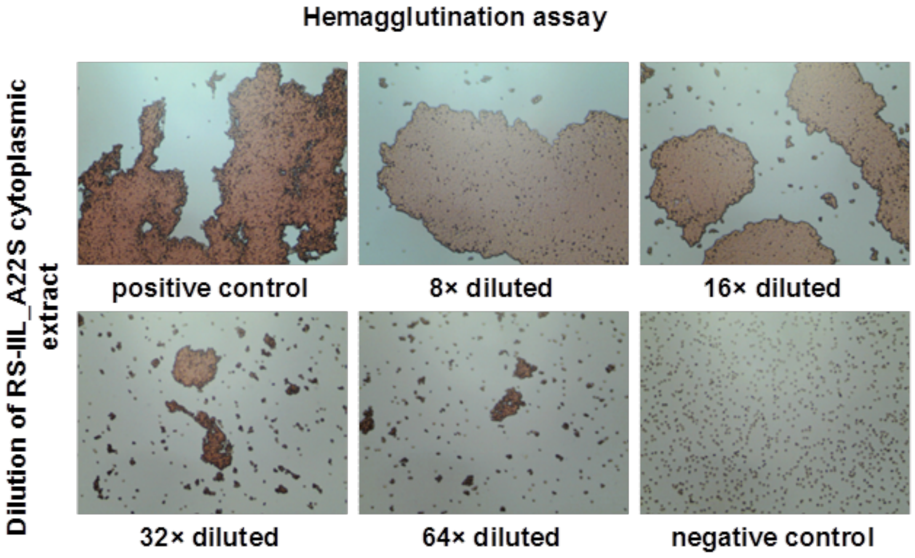

Fig. S6. Determination of cytoplasmic extract dilution appropriate for hemagglutination inhibition assays. Cytosolic extract containing CV-IIL (upper panel) or RS-IIL_A22S (lower panel) was serially diluted in working buffer and each sample from dilution line was mixed with 5% RBC_0^+ in 1 : 1 ratio. Mixture was incubated at room temperature for 5 minutes, mixed again, applied to a glass slide and observed under the Levenhuk microscope. Pictures were taken by the camera DEM135 (Levenhuk). 128× diluted cytoplasmic extract was chosen for CV-IIL and 32× diluted cytoplasmic extract was chosen for RS-IIL_A22S hemagglutination inhibition assays. All negative control experiments did not show any visible agglutination.
