# Supplementary material for: Microscopy examination of red blood and yeast cell agglutination induced by bacterial lectins
Source: PLoS One. 2019 Jul 25;14(7):e0220318. doi: 10.1371/journal.pone.0220318 (PMC6657890; doi:10.1371/journal.pone.0220318)
Supplement: S7 Fig — (PDF) [file pone.0220318.s007.pdf]

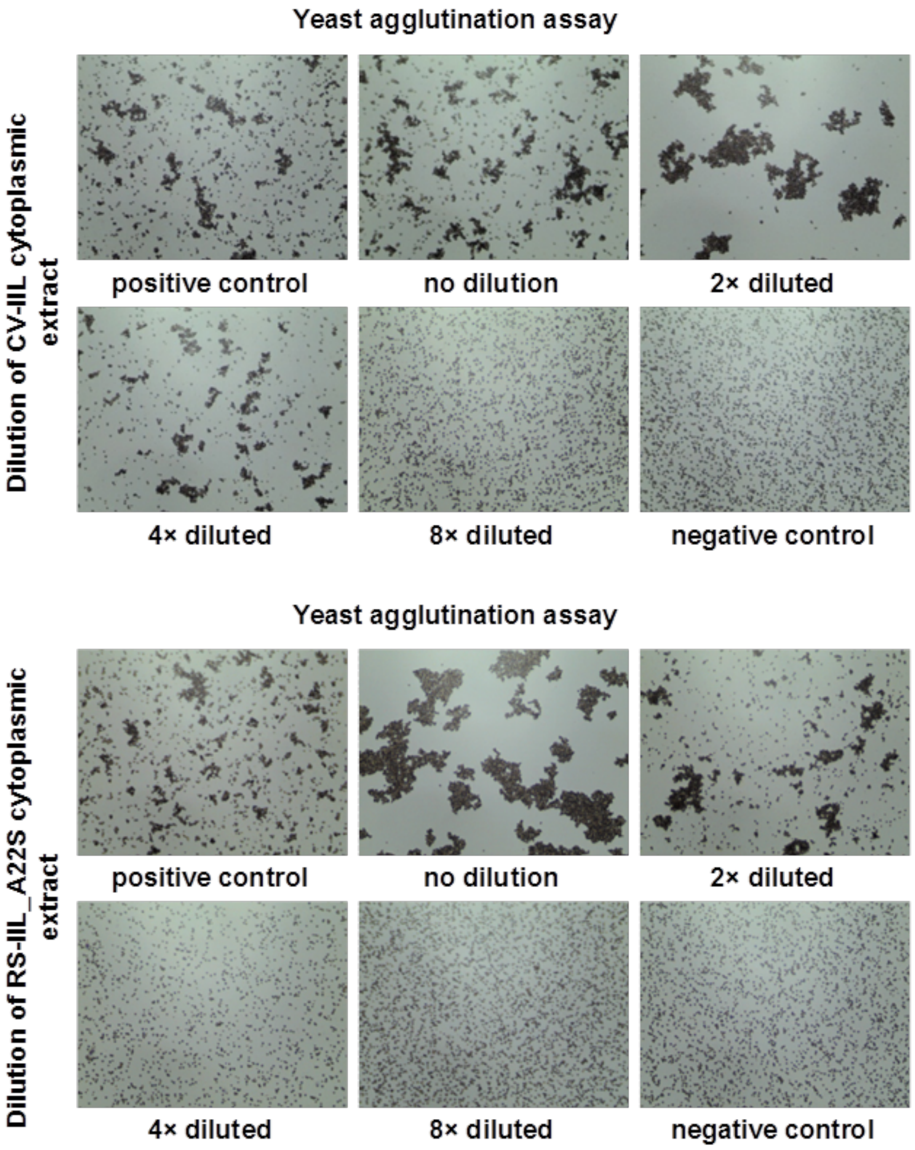

Fig. S7. Determination of cytoplasmic extract dilution appropriate for yeast agglutination inhibition assays. Cytosolic extract containing CV-IIL (upper panel) or RS-IIL_A22S (lower panel) was serially diluted in working buffer and each sample from dilution line was mixed with 5% yeast suspension in 1 : 1 ratio. Mixture was incubated at room temperature for 10 minutes, mixed again, applied to a glass slide and observed under the Levenhuk microscope. Pictures were taken by the camera DEM135 (Levenhuk). 2× diluted cytoplasmic extract was chosen for CV-IIL and undiluted cytoplasmic extract was chosen for RS-IIL_A22S yeast agglutination inhibition assays. All negative control experiments did not show any visible agglutination.
