# Supplementary material for: Microscopy examination of red blood and yeast cell agglutination induced by bacterial lectins
Source: PLoS One. 2019 Jul 25;14(7):e0220318. doi: 10.1371/journal.pone.0220318 (PMC6657890; doi:10.1371/journal.pone.0220318)
Supplement: S8 Fig — (PDF) [file pone.0220318.s008.pdf]

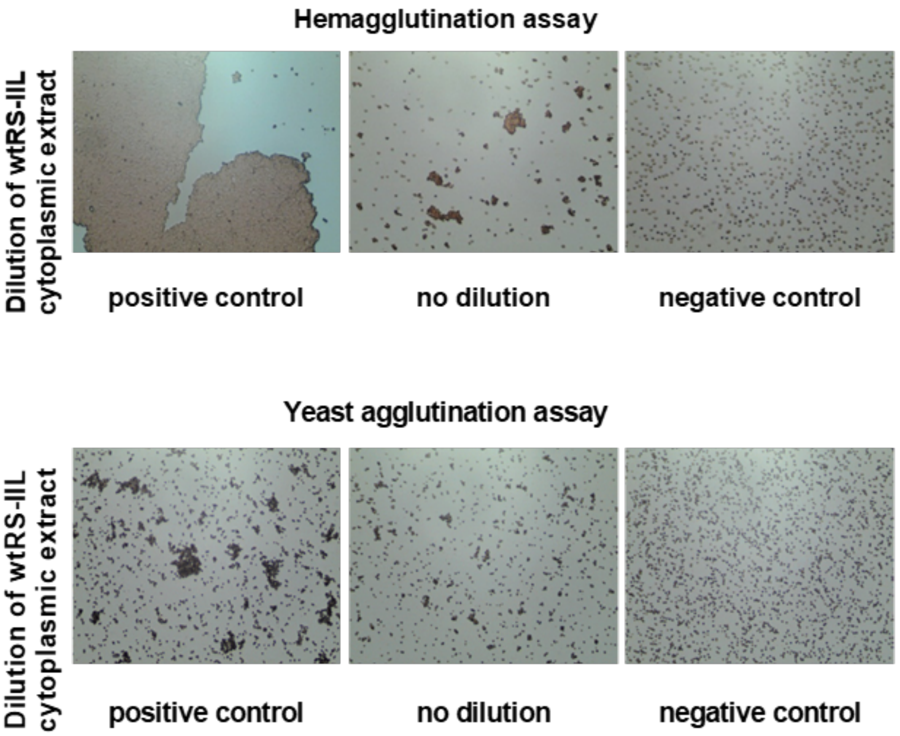

Fig. S8. Analysis of working concentration Cytosolic extract containing wtRS-IIL was mixed with 5% RBC_0$^+$ or 5% yeast suspension in 1 : 1 ratio. Mixture was incubated at room temperature for 5 minutes or 10 minutes, respectively, mixed again, applied to a glass slide and observed under the Levenhuk microscope. Pictures were taken by the camera DEM135 (Levenhuk). Undiluted cytoplasmic extract was chosen for hemagglutination and yeast agglutination inhibition assays. All negative control experiments did not show any visible agglutination.
