# Supplementary material for: Microscopy examination of red blood and yeast cell agglutination induced by bacterial lectins
Source: PLoS One. 2019 Jul 25;14(7):e0220318. doi: 10.1371/journal.pone.0220318 (PMC6657890; doi:10.1371/journal.pone.0220318)
Supplement: S9 Fig — (PDF) [file pone.0220318.s009.pdf]

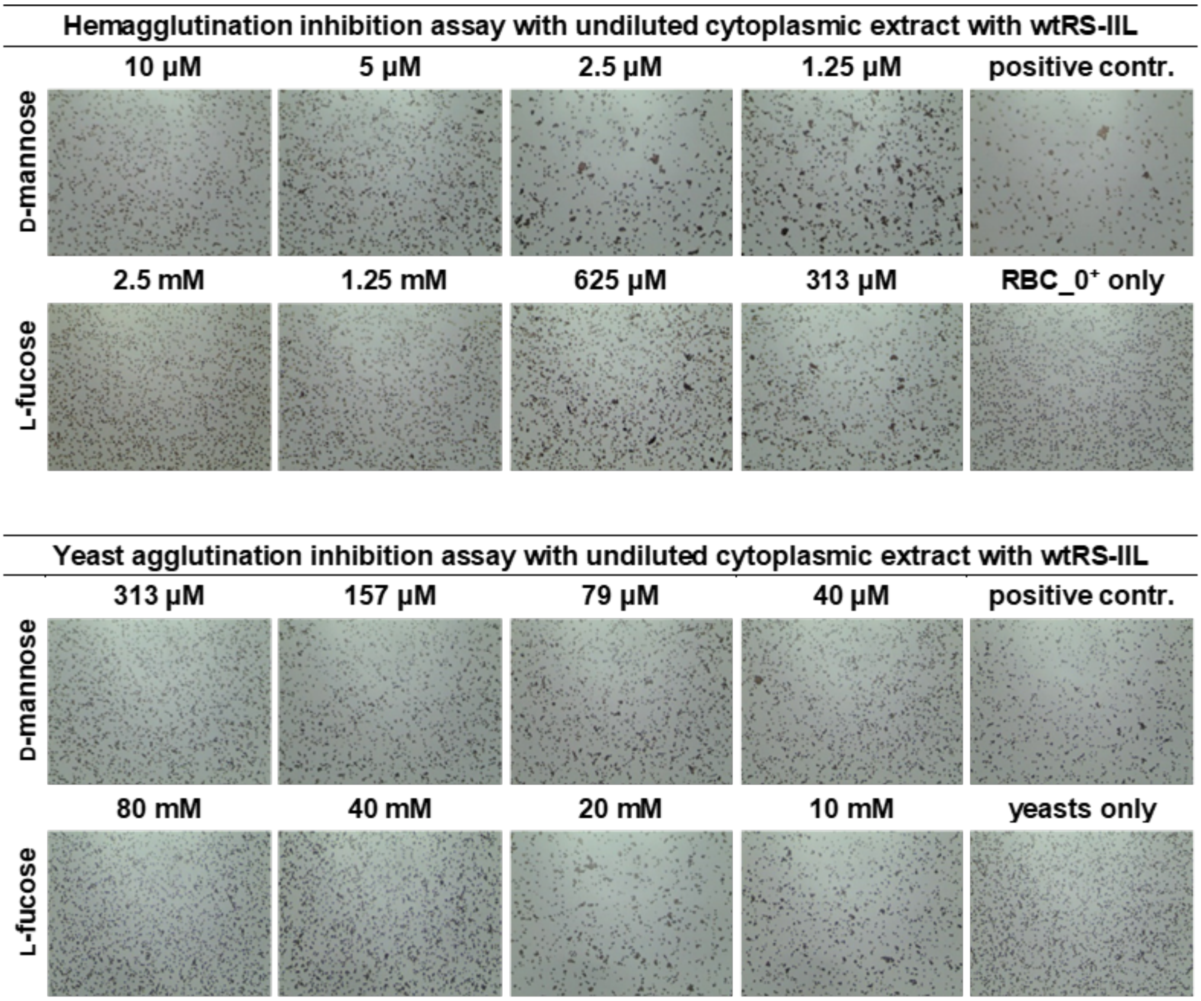

Fig. S9. Agglutination inhibition assay to test specificity of wtRS-IIL directly in *E. coli* cytosol. Hemagglutination (upper panel) and yeast agglutination inhibition assays (lower panel) with undiluted cytoplasmic extract of *E. coli* expressing *rs-2l* gene. Undiluted cytosol was mixed with the monosaccharide in a 1 : 1 ratio and incubated for 1 minute at room temperature. 10 µl of the solution was mixed with 10 µl of 5% RBC_0^+ or 5% yeast suspension and incubated at room temperature for an additional 5 (HI) or 10 (YAI) minutes, mixed again, applied to a glass slide and observed under a Levenhuk microscope. Pictures were taken with a DEM135 camera (Levenhuk).
