# Supplementary material for: Microscopy examination of red blood and yeast cell agglutination induced by bacterial lectins
Source: PLoS One. 2019 Jul 25;14(7):e0220318. doi: 10.1371/journal.pone.0220318 (PMC6657890; doi:10.1371/journal.pone.0220318)
Supplement: S10 Fig — (PDF) [file pone.0220318.s010.pdf]

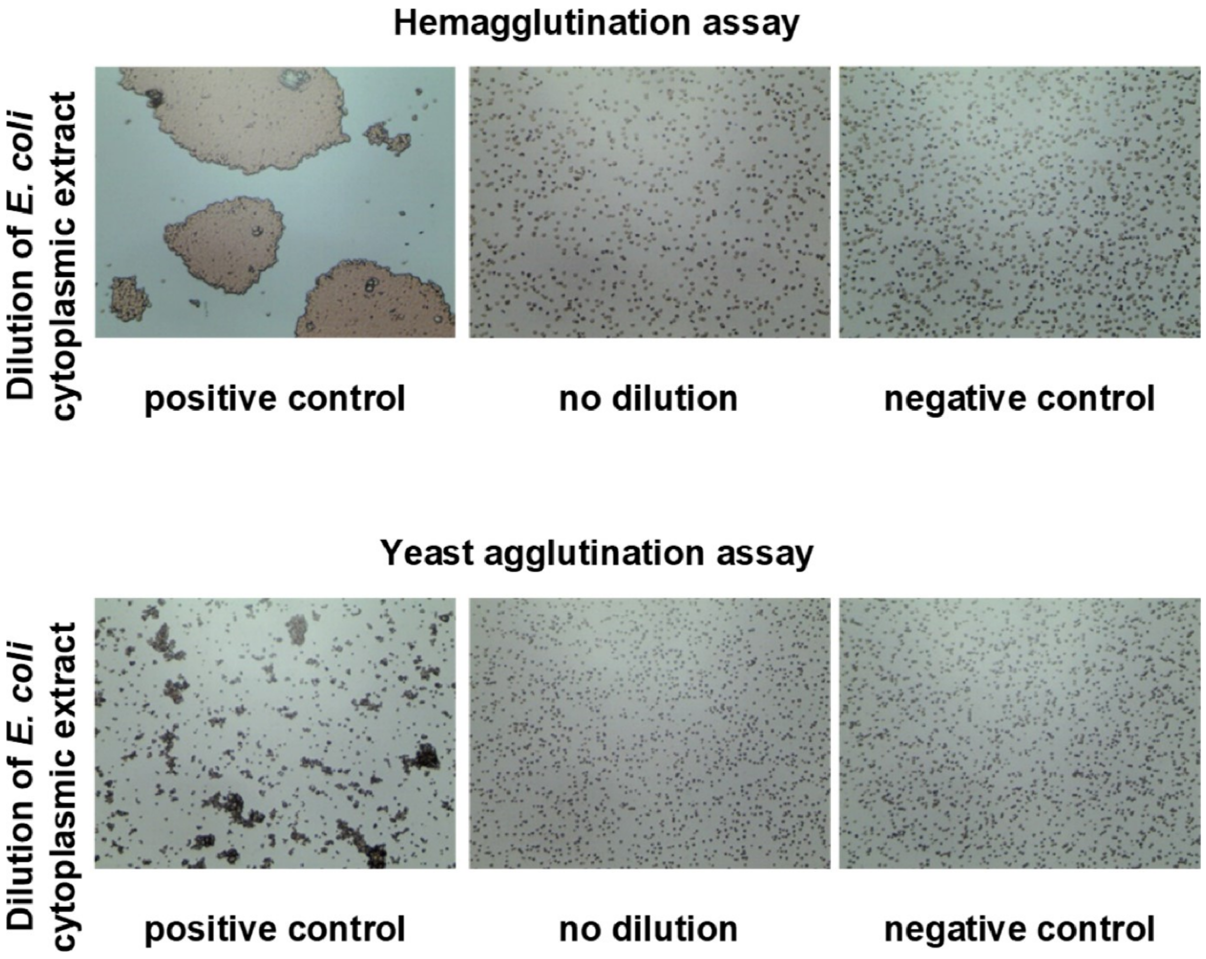

S8 Fig. Analysis of cytoplasmic extract of *E. coli* Tuner (DE3) lacking a plasmid. *E. coli* cytoplasmic extract was serially diluted in working buffer and sample of each concentration was mixed with 5% RBC_0^+ or yeast suspension in 1 : 1 ratio. Mixture was incubated at room temperature for 5 minutes or 10 minutes, respectively, mixed again, applied to a glass slide and observed under the Levenhuk microscope. Pictures were taken by the camera DEM135 (Levenhuk). No agglutination was seen in any tested dilution either in hemagglutination inhibition assay or yeast agglutination inhibition assay. CV_IIL in a concentration 0.1 mM was used as positive control. All negative control experiments did not show any visible agglutination.
