# Supplementary material for: Microscopy examination of red blood and yeast cell agglutination induced by bacterial lectins
Source: PLoS One. 2019 Jul 25;14(7):e0220318. doi: 10.1371/journal.pone.0220318 (PMC6657890; doi:10.1371/journal.pone.0220318)
Supplement: S11 Fig — (PDF) [file pone.0220318.s011.pdf]

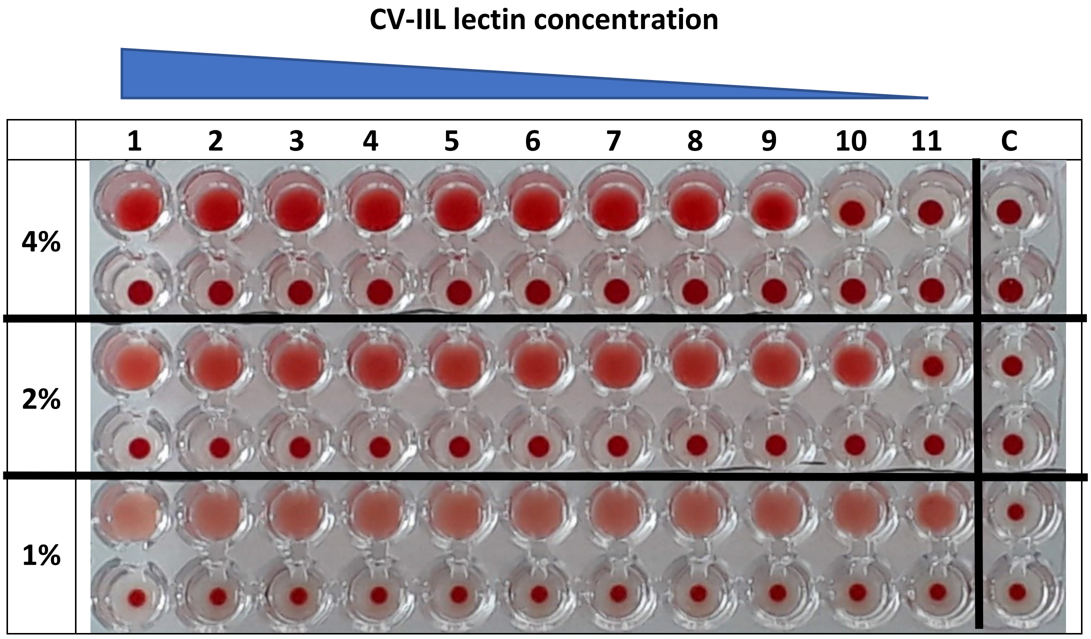

S11 Fig. Determination of RBC suspension concentration and CV-IIL titer for HI assay on microtiter plate. CV-IIL (25 µM in the first well) concentration decreases from left to right in two rows by a ratio of 0.5 between two neighboring wells. Agglutinated red blood cells form a diffuse mat, whereas non-agglutinated red blood cells sediment and form a clear dot in the bottom of the well. Three different concentration of RBC suspension (4%, 2% and 1%) were tested and CV-IIL titer was determined (well 9 in the first row of 2% RBC suspension). Last wells represent control experiments in absence of lectin.
