# Supplementary material for: Microscopy examination of red blood and yeast cell agglutination induced by bacterial lectins
Source: PLoS One. 2019 Jul 25;14(7):e0220318. doi: 10.1371/journal.pone.0220318 (PMC6657890; doi:10.1371/journal.pone.0220318)
Supplement: S12 Fig — (PDF) [file pone.0220318.s012.pdf]

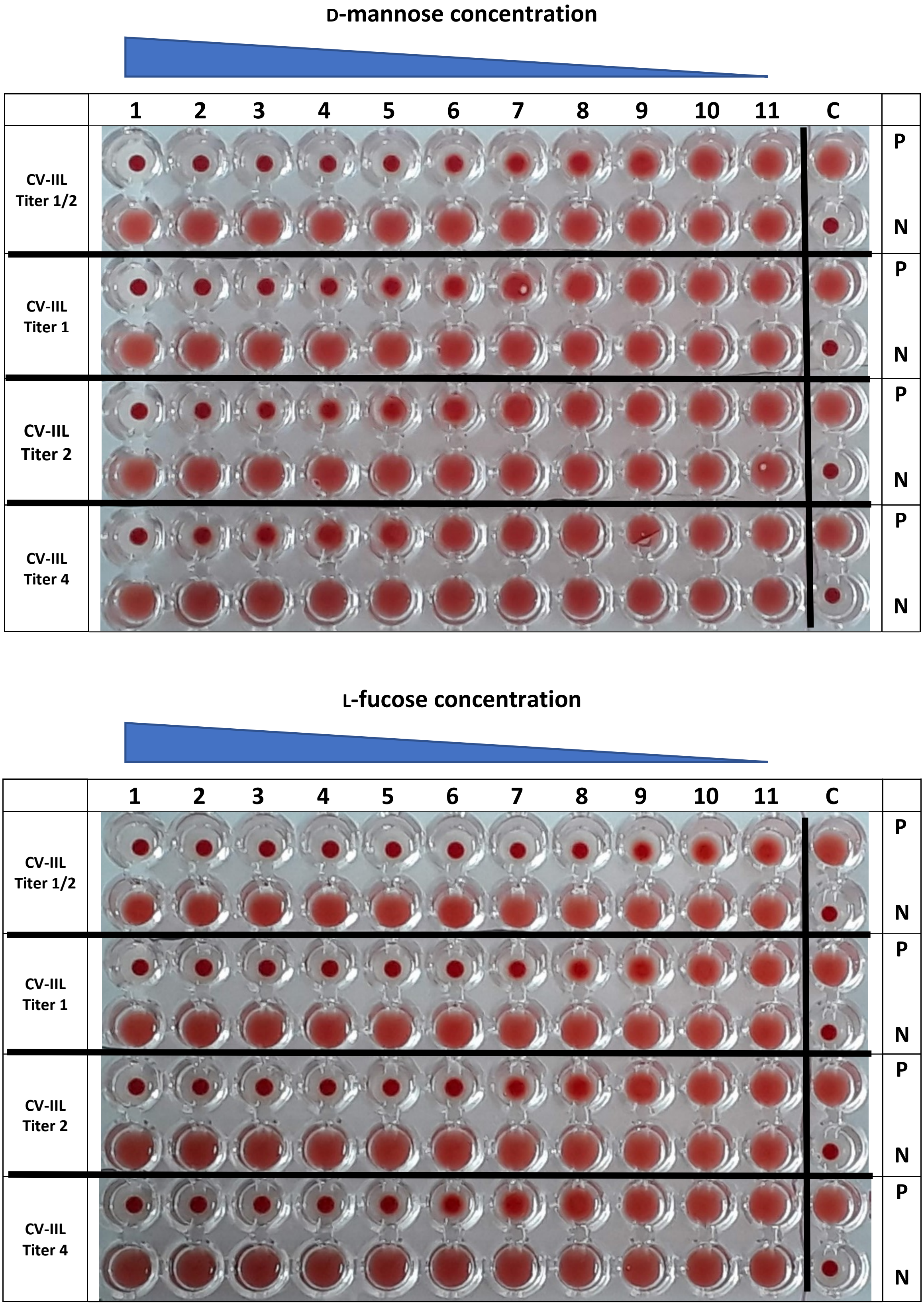

S12 Fig. Hemagglutination inhibition assay on microtiter plate with four concentrations of CV-IIL. CV-IIL in concentrations corresponding to titer ½, titer 1, 2 and 4 was tested. The first well in the first

row contains 40 mM D-mannose (upper panel) or L-fucose (lower panel). Carbohydrate concentration decreases from left to right in two rows by a ratio of 0.5 between two neighboring wells. Inhibited agglutination results in a clear dot in the bottom of the well, whereas non-inhibited agglutination results in a diffuse mat. Last wells represent control experiments. In positive controls (P) buffer was used instead of monosaccharide. In a negative controls (N) buffer was used instead of lectin.
