# Supplementary material for: Microscopy examination of red blood and yeast cell agglutination induced by bacterial lectins
Source: PLoS One. 2019 Jul 25;14(7):e0220318. doi: 10.1371/journal.pone.0220318 (PMC6657890; doi:10.1371/journal.pone.0220318)
Supplement: S14 Fig — (PDF) [file pone.0220318.s014.pdf]

	1 hour of incubation	negative control
The titer of CV-IIL	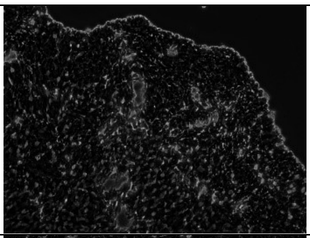	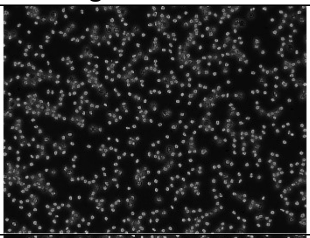
The titer of RS-IIL_A22S	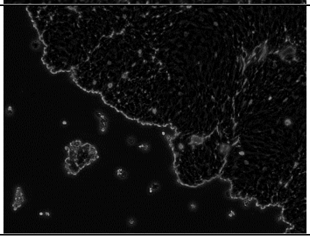	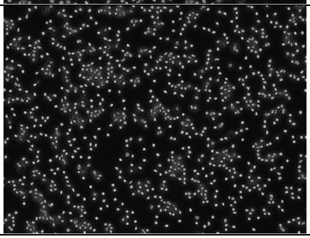

S14 Fig. RBC condition after 1 hour of incubation with CV-IIL and RS-IIL_A22S in concentration of the titer. Agglutination assays with serially diluted CV-IIL or RS-IIL_A22S were done. The last sample of each protein which showed total agglutination (the titer) was investigated using motorized inverted fluorescence microscope IX81 (Olympus). Pictures were captured at 200× magnification. Negative control (working buffer used instead of the lectin) did not show any agglutination.
